# Aberrant palmitoylation caused by a ZDHHC21 mutation contributes to pathophysiology of Alzheimer’s disease

**DOI:** 10.1186/s12916-023-02930-7

**Published:** 2023-06-26

**Authors:** Wenwen Li, Yana Pang, Yan Wang, Fan Mei, Mengmeng Guo, Yiping Wei, Xinyue Li, Wei Qin, Wei Wang, Longfei Jia, Jianping Jia

**Affiliations:** 1 Innovation Center for Neurological Disorders and Department of Neurology, Xuanwu Hospital, Capital Medical University, National Clinical Research Center for Geriatric Diseases, Beijing, China; 2 Institute of Systems Biomedicine, Department of Pathology, School of Basic Medical Sciences, Peking University Health Science Center, Beijing, China; 3 Beijing Key Laboratory of Geriatric Cognitive Disorders, Beijing, China; 4 Clinical Center for Neurodegenerative Disease and Memory Impairment, Capital Medical University, Beijing, China; 5 Center of Alzheimer’s Disease, Beijing Institute of Brain Disorders, Collaborative Innovation Center for Brain Disorders, Capital Medical University, Beijing, China; 6 Key Laboratory of Neurodegenerative Diseases, Ministry of Education, Beijing, China

**Keywords:** ZDHHC21, Palmitoylation, Synaptic dysfunction, FYN, Alzheimer’s disease

## Abstract

**Background:**

The identification of pathogenic mutations in Alzheimer’s disease (AD) causal genes led to a better understanding of the pathobiology of AD. Familial Alzheimer’s disease (FAD) is known to be associated with mutations in the *APP, PSEN1,* and *PSEN2* genes involved in Aβ production; however, these genetic defects occur in only about 10–20% of FAD cases, and more genes and new mechanism causing FAD remain largely obscure.

**Methods:**

We performed exome sequencing on family members with a FAD pedigree and identified gene variant ZDHHC21 p.T209S. A ZDHHC21^T209S/T209S^ knock-in mouse model was then generated using CRISPR/Cas9. The Morris water navigation task was then used to examine spatial learning and memory. The involvement of aberrant palmitoylation of FYN tyrosine kinase and APP in AD pathology was evaluated using biochemical methods and immunostaining. Aβ and tau pathophysiology was evaluated using ELISA, biochemical methods, and immunostaining. Field recordings of synaptic long-term potentiation were obtained to examine synaptic plasticity. The density of synapses and dendritic branches was quantified using electron microscopy and Golgi staining.

**Results:**

We identified a variant (c.999A > T, p.T209S) of *ZDHHC21* gene in a Han Chinese family. The proband presented marked cognitive impairment at 55 years of age (Mini-Mental State Examination score = 5, Clinical Dementia Rating = 3). Considerable Aβ retention was observed in the bilateral frontal, parietal, and lateral temporal cortices. The novel heterozygous missense mutation (p.T209S) was detected in all family members with AD and was not present in those unaffected, indicating cosegregation. ZDHHC21^T209S/T209S^ mice exhibited cognitive impairment and synaptic dysfunction, suggesting the strong pathogenicity of the mutation. The ZDHHC21 p.T209S mutation significantly enhanced FYN palmitoylation, causing overactivation of NMDAR2B, inducing increased neuronal sensitivity to excitotoxicity leading to further synaptic dysfunction and neuronal loss. The palmitoylation of APP was also increased in ZDHHC21^T209S/T209S^ mice, possibly contributing to Aβ production. Palmitoyltransferase inhibitors reversed synaptic function impairment.

**Conclusions:**

ZDHHC21 p.T209S is a novel, candidate causal gene mutation in a Chinese FAD pedigree. Our discoveries strongly suggest that aberrant protein palmitoylation mediated by ZDHHC21 mutations is a new pathogenic mechanism of AD, warranting further investigations for the development of therapeutic interventions.

**Supplementary Information:**

The online version contains supplementary material available at 10.1186/s12916-023-02930-7.

## Background

Alzheimer’s disease (AD) is a devastating neurodegenerative disease that afflicts a large portion of the older population at an ever-increasing rate. Synaptic dysfunction, neuronal loss, amyloid plaques, neurofibrillary tangles, brain atrophy, and cognitive impairment are the pathological and clinical features of AD [1–4]. Mutations in genes involved in amyloid beta (Aβ) production, such as Aβ precursor protein (*APP*), presenilin-1 (*PSEN1*), and presenilin-2 (*PSEN2*), are known to play major roles in certain cases of familial AD (FAD) [5–8]. The identification of causal gene mutations has greatly improved our understanding of the genetic mechanisms underlying AD. However, most of these pathogenic mutations display an autosomal-dominant pattern of inheritance and occur in a low proportion (< 5%) of patients with AD [9, 10]. Recently, the Chinese Familial Alzheimer’s Disease Network (CFAN) was established to study the genetic features of Chinese individuals with FAD [11]. In total, 1330 patients with AD or mild cognitive impairment in 404 pedigrees were enrolled; among them, 13.12% of pedigrees carried *PSEN/APP* missense mutations, 3.71% carried *PSEN/APP* synonymous/untranslated region variants, and 83.17% did not carry such mutations or variants [11]. This low mutation rate suggests that other genes and mechanism may be involved in FAD.

In addition to causal variants, variants in more than 600 genes have been investigated as susceptibility factors for AD, including *APOE*, *TREM2*, *SORL1*, and *ABCA7*. The diversity of cellular functions implicated by the growing list of risk variants associated with AD underscores the pathogenic heterogeneity of the disease. All such results add to the knowledge of the pathogenic mechanisms that drive the development of AD. However, to date, results of clinical trials in AD have been disappointing. Thus, more causal and/or risk-related gene variants and new mechanisms need to be uncovered to improve the understanding of the complex pathogenesis of AD.

In this study, we identified a rare gain-of-function variant, p.T209S, in the zinc finger DHHC-type palmitoyltransferase 21 (*ZDHHC21*) gene on chromosome 9 that segregated with the disease in an autosomal-dominant pattern in a Chinese family with FAD. We constructed ZDHHC21 p.T209S mice by using the CRISPR/Cas9 system to characterize the pathogenesis related to this variant. We discovered that ZDHHC21 p.T209S contributed to AD by enhancing the palmitoylation of FYN and APP, resulting in AD pathology and synaptic dysfunction, ultimately causing cognitive impairment. Furthermore, we revealed that palmitoylation inhibitors rescued the impaired synaptic function. Our results indicate that palmitoylation modification of specific substrates is an alternative driving mechanism of AD and suggest that palmitoylation is a promising target for drug therapy.

## Methods

### Antibodies

The following antibodies were used in this study: anti-Fyn (catalogue no. ab1881, Abcam), anti-Fyn (catalog no.: sc-434, Santa Cruz Biotechnology), anti-APP (catalog no.: ab32136, Abcam), anti-actin (catalog no.: TA-09, Zsgb-Bio, China), anti-NMDAR2B (catalog no.: ab254356, Abcam), anti-ZDHHC21 (catalog no.: PA5-25,096, Invitrogen, USA), anti-NeuN (catalog no.: ab279295, Abcam), anti-tau (phospho T231; catalog no.: ab151559, Abcam), anti-tau (phospho Thr181; catalog no.: 701530, Invitrogen), anti-Aβ (catalog no.: ab201060, Abcam), anti-phospho-Src family (catalog no.: 2101S, Cell Signaling Technology, USA), horseradish peroxidase (HRP)-conjugated goat anti-rabbit IgG heavy chain-specific secondary antibody (catalog no.: A25222, Abbkine, China), HRP-conjugated mouse anti-rabbit IgG light chain (catalog no.: A25022, Abbkine), goat anti-mouse IgG H&L (HRP; catalog no.: ab6789, Abcam), goat anti-rabbit IgG H&L (HRP; catalog no.: ab205718, Abcam), goat anti-rabbit IgG H&L (Cy3; catalog no.: ab6939, Abcam), and goat anti-mouse IgG H&L (FITC; catalog no.: ab6785, Abcam).

### Patients and ethical considerations

We recruited patients with a positive family history from the CFAN [11]. In two generations of this family, three family members (I-2, II-2, and II-6) had AD, and II-6 was the proband. She was evaluated using the MMSE, the CDR scale, CT, and molecular neuroimaging with PET. The MMSE is used as the gold standard for cognitive deficit screening, with a total score ranging from 0 to 30, with lower scores reflecting worse impairment. The global CDR score ranges from 0 (healthy) to 3 (severe dementia) [12]. N-methyl-[^11^C]2-(4’-methylaminophenyl)-6-hydroxybenzothiazole (^11^C-PIB) positron emission tomography (PET) was used to measure Aβ retention. [13]. The diagnostic criteria for AD were mainly based on recommendations of the National Institute on Aging and Alzheimer's Disease [14]. The institutional review board of Xuanwu Hospital, Capital Medical University, approved this study, and all participants provided written informed consent.

### Gene sequencing and cosegregation analysis

Whole exome sequencing was performed on the patients to identify pathogenic genes. Briefly, genomic DNA was extracted from peripheral blood and fragmented into 200–300-bp fragments. DNA libraries were prepared using a KAPA Library Preparation Kit (catalog no.: KR0453, Kapa Biosystems, USA). The amplified DNA sample was captured using the Agilent SureSelectXT2 Human All Exon V6 kit (Agilent Technologies, USA), and sequenced on the Illumina NovaSeq S4 platform (Illumina, USA) with 150-bp paired-end reads, according to the standard manual. Raw data were filtered and aligned against the human reference genome (UCSC hg19) by using the Burrows-Wheeler Alignment tool (BWA-0.7.12, http://bio-bwa.sourceforge.net/). GATK software (www.broadinstitute.org/gatk) was used to call single-nucleotide polymorphisms, insertions, and deletions. The Ensembl Variant Effect Predictor was used to annotate all variants for position, type, allele frequency, conservation prediction, etc. [15]. We used the HGMD (https://www.hgmd.cf.ac.uk/) to confirm previously reported pathogenic mutations. Multiple software programs, such as SIFT, PolyPhen-2, and Mutation Taster, were used to predict the impact of the variants. All candidate pathogenic variants were confirmed via Sanger sequencing and classified according to the American College of Medical Genetics and Genomics standards. Cosegregation analysis was performed in light of previous methods [16].

### Generation of ZDHHC21 p.T209S mutant mouse model

Mice with c.999A > T point mutations in *Zdhhc21* were designed and generated by Shanghai Model Organisms Center, Inc. (China). Briefly, Cas9 mRNA was transcribed in vitro using the mMESSAGE mMACHINE T7 Ultra Kit (catalog no.: AM1344, Ambion, USA), according to the manufacturer’s instructions, and subsequently purified using the MEGAclear™ Kit (catalog no.: AM1908, Thermo Fisher Scientific, USA). 5'-TCAAAGGTAGTCAAACAGTATGG-3' was chosen as the Cas9 targeted single guide RNA (sgRNA), transcribed in vitro using the MEGAshortscript Kit (catalog no.: AM1345, Thermo Fisher Scientific), and subsequently purified using a MEGAclear™ Kit. The transcribed Cas9 mRNA and sgRNA, as well as 224 bp of single-stranded oligodeoxynucleotides (ssODNs), were co-injected into zygotes of C57BL/6 J mice. The F0 mice were validated via PCR and sequencing with the following primer pairs: F1: 5’-ACACTCTGAAGACTGTTGTTGGAAGC-3’ and R1: 5’GCACTGTAGATATGAAGGGGTTGC-3’. F0 mice with the expected point mutations were chosen and crossed with C57BL/6 J mice to produce F1 mice. The genotypes of the F1 mice were identified via PCR and confirmed via sequencing. The sequence of the ssODN for the mice with point mutations was as follows:

5'-AACTTGTTAAAAGTATTTATAAATTTGTTACATCTGAATATAATTTTTTGTTTTTAATAGGATTCAACATCTATTGAAAAAATGTCAAATTGTTGTGAAGAAATGTAAGTATTAAGTTATAGTGATCTTATTTAAAAATAGAAACGATACTGTTTGACTACCTTTGAGAATCATGTGCCTATGTTAATAGTAGGAAACTAATATGTTGATTTTTTTTTCTTGGT-3’.

The Specific pathogen Free environment was required for the housing and husbandry conditions of mice in this study (Approval Number: Medical document No. 01–4058).

### Culture of mouse primary cortical neurons

Primary neurons were obtained on embryonic days 15–16 from C57BL/6 J WT and ZDHHC21^T209S/T209S^ pregnant mice. The meninges and vascular membranes were quickly removed, and the cerebral cortex tissues were digested with TrypLE Select Enzyme (catalog no.: 12563011, Gibco, USA) for 10 min and subsequently added to complete Dulbecco’s Modified Eagle Medium with 10% fetal bovine serum and 0.1 mg/ml DNase I (catalog no.: 10104159001, Sigma, Germany). This solution was mechanically separated 30 times to gain a cell suspension, and the cells were cultured in poly-D-lysine-coated (catalog no.: P6407, Sigma) 6-well plates at a density of 1 × 10^6^ cells/well in a neurobasal medium (catalog no.: 21103049, Gibco) with 2% B27 supplement (catalog no.: 17504044, Gibco) and 1% glutamine (catalog no.: 35050061, Gibco).

### Morris water maze task

The Morris water maze test was performed as described in our previous experiments, with slight modification [17]. On the first day of training, the platform was located 1 cm above the water surface, and a small flag was placed on the platform to increase recognition. Subsequently, mice were trained to find a platform hidden 1 cm below the water surface for four consecutive days. The complete training process lasted five days, including four tests per day, using different entry points (up to 60 s; 30-min intervals). The probe trial was performed on day 6. We calculated the number of times each mouse crossed the platform position and the time spent in the quadrant where the platform was located. The trajectory was recorded by using the Smart 3.0 video tracking system (Panlab, Spain).

### Western blotting

Western blotting was performed according to our previous protocol, with minor changes [18, 19]. Briefly, for total protein extraction, cells and tissues were lysed in RIPA lysis buffer (catalog no.: C1053, Applygen, China) with 100 W ultrasound for 3 min, in which the ultrasonic time was 10 s and the rest time was 30 s, repeated three times. The samples were subsequently centrifuged at 12,000 × g for 20 min, after which the protein supernatant was absorbed. For membrane proteins, we strictly followed the Mem-PER Plus Membrane Protein Extraction Kit (catalog no.: P0033, Beyotime, USA). First, we took approximately 100 mg of mouse brain tissue and carefully cut it into tiny fragments with scissors. Thereafter, we added 1 ml of membrane protein extraction reagent A with phenylmethylsulphonyl fluoride (1 mM), gently suspended the tissue fragments in an ice bath for 10–15 min, transferred the tissue sample to a pre-chilled glass homogenizer for approximately 30–50 rounds of homogenization, and centrifuged it at 4 °C and 700 × g for 10 min. We carefully collected the supernatant into a fresh centrifuge tube to remove nuclei and unbroken cells and centrifuged it at 4 °C and 14,000 × g for 30 min to pellet cell membrane debris. Thereafter, we added 200 μl of membrane protein extraction reagent B and vortexed the solution and incubated it in an ice bath 15 times to adequately extract membrane proteins. Finally, we centrifuged the solution at 4 °C and 14,000 × g for 5 min and collected the supernatant as the cell membrane protein solution. Protein concentration was measured via the BCA method, and we used the SDS protein-loading method for denaturation. The protein samples were loaded for SDS/PAGE.

### Co-immunoprecipitation

Proteins were obtained from tissues lysed in lysis buffer (30 mM HEPES, 100 mM NaCl, 0.5% Nonidet P-40, protease inhibitor mixture, pH: 7.6) on ice for 30 min, followed by centrifugation at 13,000 rpm for 10 min. Immunoprecipitation was performed with 500 μg protein in the presence or absence of APP/FYN-specific antibodies (1:200, Abcam, UK). After incubation for 3 h, protein A/G agarose beads were added for 1 h and pulled down. The immune complexes were washed three times with phosphate-buffered saline (PBS), eluted with 2 × sample buffer, and subjected to 10% SDS-PAGE. We used 1% (relative to that used for immunoprecipitation) of the lysate as input. We analyzed the presence of relevant proteins in immune complexes via western blotting using anti-ZDHHC21 (1:200, Thermo Fisher Scientific) antibodies.

### Immunofluorescent staining

We used 4-μm paraffin-embedded sections of the mouse brain for immunostaining, first permeating and dehydrating it in xylene and gradient anhydrous ethanol, followed by immune antigen repair with sodium citrate (pH: 6.0) or Tris–EDTA (pH: 9.0). Subsequently, the primary anti-ZDHHC21 (1:50, Thermo Fisher Scientific) and anti-FYN (1:20, Santa Cruz Biotechnology, USA) antibodies were cultured at 4℃ overnight, and incubated with fluorescently labeled secondary antibodies (Cy3-labeled ZDHHC21, 1:100, Abcam; fluorescein isothiocyanate [FITC]-labeled FYN, 1:100, Abcam) for 2 h at 37℃. Anti-NeuN and TUNEL co-staining was performed: 40 μl TUNEL detection solution (catalog no.: 11684817910, Roche, Switzerland) was added to the sections, the sections were incubated at 37 °C for 2 h, and the sections were incubated overnight with anti-NeuN at 4℃. Sections were incubated in saline with a mixture of Cy3-labeled second-generation antibodies (1:300, Abcam), and DAPI (catalog no.: 40728ES03, Yeasen, China) was used for nuclear staining. Brain sections were sealed with an anti-fluorescence quenching sealing solution and imaged using a laser confocal microscope (Zeiss LSM 980, Zeiss, Germany).

### Immunohistochemistry

After perfusion with 0.01 M PBS and dissection, the left hemibrain of each mouse was fixed with 4% paraformaldehyde, dehydrated with ethanol and xylene, and embedded in paraffin for preservation. Brain tissues were sectioned with a paraffin microtome to a thickness of 5 μm and mounted on poly-L-lysine-coated slides. Antigen retrieval was performed using 10 mM sodium citrate (pH: 6.0) and 15 min of microwaving. After being washed with PBS, the sections were pre-treated with 3% H_2_O_2_/3% methanol in PBS for 10 min to block endogenous peroxide activity. For Aβ, the sections were blocked in PBS containing 10% normal goat serum for 30 min at 37℃, and subsequently incubated with anti-Aβ (1:2000, Abcam) for 1 h at 37℃. The sections were incubated with biotin-labeled secondary antibodies (goat anti-rabbit IgG [H&L], 1:2000, Abcam) for 30 min at room temperature, washed with PBS, and incubated with the ABC kit (catalog no.: PK-6100, Vectastain, China). For p-tau, the sections were blocked in PBS containing 3% bovine serum albumin for 30 min at 37℃, and incubated with anti-tau (phospho T231, 1:500, Abcam) for 1 h at room temperature. The sections were incubated with horseradish enzyme-labeled secondary antibodies (goat anti-rabbit IgG [H&L], 1:2000, Abcam) for 30 min at room temperature. Positive signals were visualized using a DAB kit (catalog no.: DA1010, Solarbio, China). The sections were stained with Mayer’s hematoxylin solution (catalog no.: G1080, Solarbio) for 30 s, dehydrated in ethanol and xylene, mounted with neutral gum, and imaged with a microscope (Olympus CX33, Olympus, Japan).

For Aβ and p-tau quantification, 16 bit images were converted to 8 bit gray-scale images. After defining the region of interest (hippocampal CA1 and DG), they were thresholded within a linear range using NIH ImageJ software (https://imagej.nih.gov/ij/) [20]. The images were then adjusted to the same threshold to increase the signal-to-noise ratio. Four sections per mouse were quantified. The quantification of WT and ZDHHC21^T209S/T209S^ mice was performed in a blinded manner.

### ABE palmitoylation assay

This method is based on a previously described ABE assay with slight modification [21]. Briefly, hippocampal tissues of 6-month-old mice were lysed in RIPA buffer containing protease and phosphatase inhibitors to adjust the protein concentration to 1.0 μg/µl. At least 500 μg of total protein in 500 μl lithium borate buffer was precipitated with chloroform–methanol before 10 mM N-ethylmaleimide treatment at 4℃ overnight. After three rounds of precipitation with chloroform–methanol, palmitate was cleaved from cysteines on the protein with hydroxylamine (HA), and the absence of HA cleavage served as a negative control. All proteins were nutated for 45 min at room temperature. Thereafter, 1 μM of biotin-BMCC was used to label the free thiol groups of cysteines on the proteins, with 45 min of nutation at 4 °C. The biotin-conjugated target protein was captured using streptavidin-agarose beads and eluted with SDS sample buffer containing β-mercaptoethanol. Palmitoylation of the target protein was detected via western blotting with the corresponding antibody.

### ELISA

Aβ ELISA kits were used to measure Aβ42 (catalog no.: CSB-E10787m, Cusabio, China) and Aβ40 (catalog no.: CSB-E08300m, Cusabio) levels. In short, the tissues were ultrasonically lysed in PBS liquid and centrifuged at 12,000 rpm at 4 °C for 20 min. The supernatant of the lysate was transferred to a 96-well plate. It was incubated with the secondary antibody at room temperature for 1 h, and the optical density was measured at 450 nm.

### Golgi staining

Golgi staining was conducted in strict accordance with the manufacturer’s information via the FD Rapid GolgiStain Kit (catalog no.: PK-401, FD NeuroTechnologies, USA). Briefly, solutions A and B were mixed 24 h in advance. The tissue was immersed in that mixture for 2 weeks, transferred to solution C for 5 days, immersed in isopentane liquid (catalog no.: M108172; Aladdin, China), mixed with dry ice for freezing, embedded with a freezing embedding agent (catalog no.: TFM-5, Sakura, USA), and frozen at − 80℃. Thereafter, the tissues were cut into sections of 100 μm for silver staining and dehydrated in gradient alcohol. The background color was removed in xylene liquid and the sections were sealed with gum (catalog no.: 36313ES60, Yeasen). The tissue sections were observed under a forward fluorescence microscope (Nikon DS-Ri2, Nikon, Japan).

For statistical analysis of dendritic spines and branches, we used Sholl analysis [22] (via the FIJI image processing package). First, we converted the image into 8 bits, set the scale, and saved it. Second, we opened the image, tracked the trajectory of the neuron, measured the length of the neuron, and performed neuron tracing with the plugin “neuron J.” Finally, we used the plugin “sholl analysis” to form concentric circles of isometric distance around the neuron. We calculated the number of intersections of dendrites and concentric circles, as such intersections indirectly reflect the length of the dendrites and the number of branches.

### Transmission electron microscopy

According to a published protocol [23], after anesthesia, mice were injected into the left ventricle with 4% paraformaldehyde. Hippocampal sections were fixed in cold 1% OsO_4_ for 1 h. The specimens were prepared and tested according to standard procedures. Ultrathin Sects. (90 nm) were stained with uranyl acetate and lead acetate and observed under a transmission electron microscope (Tecnai G2 20 Twin, FEI, USA). Synapses were identified based on synaptic vesicles and postsynaptic density.

### RNA isolation and quantitative reverse transcription-PCR analysis

RNA was extracted as previously described [18], using the Total Cell/Tissue RNA Isolation Kit (catalog no.: RC112-01, Vazyme, China), and reverse-transcribed to cDNA by using the HiScript II Q Select RT SuperMix (catalog no.: R233-01, Vazyme). Quantitative PCR (qPCR) was performed using the AceQ Universal SYBR qPCR Master Mix (catalog no.: Q511-02, Vazyme) on an Applied Biosystems instrument (Thermo Fisher Scientific, USA). The qPCR protocol was as follows: 40 cycles of 95 °C for 5 min, 95 °C for 10 s, and 60 °C for 30 s. Relative expression was calculated with the 2-^ΔΔCT^ method [24].

The ZDHHC21 primer pairs were as follows: F: 5’-ATGGGTCTTCGGATTCACTTTG-3’, R: 5’-CCCTCACTAAGGCAACCAGG-3’.

### Electrophysiology

Three-month-old male mice were anesthetized by intraperitoneal injection of pentobarbital (0.05 g/kg body weight) and decapitated. Coronal hippocampal Sects. (400 µm) were prepared using a vibrating microtome (Leica VT2000S, Leica Biosystems, Germany) in ice-cold slicing buffer aerated with 95% O_2_ and 5% CO_2_ (26 mM NaHCO_3_, 1.25 mM NaH_2_PO_4_, 2.5 mM KCl, 0.5 mM CaCl_2_, 4 mM MgCl_2_, 10 mM dextrose, and 206 mM sucrose). Sections were submerged in oxygenated artificial cerebrospinal fluid (ACSF; 125 mM NaCl, 26 mM NaHCO_3_, 1.25 mM NaH_2_PO_4_, 2.5 mM KCl, 2 mM CaCl_2_, 1 mM MgCl_2_, and 10 mM dextrose) for 1 h at room temperature. Recordings were acquired using a MultiClamp 700B microelectrode amplifier and Digidata 1440A low-noise data acquisition system (Molecular Devices, USA). fEPSPs were evoked in the CA1 stratum radiatum by stimulating the Schaffer collateral with a bipolar tungsten electrode and recorded with ACSF-filled glass pipettes (resistance < 1 MΩ) at 30 °C. Baseline responses were set to 40% of the maximal response and were recorded for 15 min. Long-term potentiation was induced by one 100 Hz tetanus (1 s). The data were analyzed using Clampfit 10 software (Molecular Devices). The peak amplitude of the fEPSP was measured as an index of synaptic strength and expressed as a percentage of the baseline average.

### NMDA-mediated excitotoxicity assays

We measured the impact of palmitoylation on cell viability following exposure of WT and ZDHHC21^209S/T209S^ neurons to NMDA and glycine by plating and pretreating primary cortical neurons with dimethyl sulfoxide (vehicle), 1 µM 2-BP (catalog no.: 238422, Sigma) or 1 µM cerulenin (catalog no.: C102399, Aladdin) between DIV11 and DIV18. Subsequently, feeding medium was removed from the neurons, stored at 37℃, and replaced with B27 neurobasal medium with or without NMDA/glycine at concentrations of 100/10 µM, respectively. After incubation at 37℃ for 2 h, the treatment medium was removed and replaced with the original feeding medium. The cells were incubated for an additional 22 h. Cell viability was assayed using the PrestoBlue HS cell viability reagent (catalog no.: P50200, Thermo Fisher Scientific) and measured using a Thermo Fisher Multiskan GO Multimode Detector, according to the manufacturer’s instructions. Cell viability was calculated as a percentage of vehicle-treated control cells.

### Calcium imaging analyses

For direct imaging of calcium signaling in the primary neurons of WT and ZDHHC21^209S/T209S^ mice, a construct encoding GCaMP3 (catalog no.: Plasmid 22,692, Addgene, USA) was introduced into the cells at DIV8. One group of cells was treated with 1 µM 2-BP (catalog no.: 10005647, Cayman Chemicals, USA) between DIV12 and DIV18. Cells were cultured to DIV18 and imaging was performed for up to 15 min in Tyrode’s solution (imaging medium, 139 mM NaCl, 3 mM KCl, 17 mM NaHCO_3_, 12 mM glucose, and 3 mM CaCl_2_) by using a camera mounted on a laser confocal microscope (Zeiss LSM 980, Zeiss). Each neuron was recorded for up to 5 min (at least three neurons per coverage; *n* = 3–6 neurons/group, three independent experiments). When Ro 25–6981 was processed, neurons were imaged at baseline for an initial 2–2.5 min; thereafter, Ro 25–6981 (1 µM) was added directly to the imaging medium before the neurons were imaged for an additional 2.5 min. p.T209S primary neurons at DIV12-18 (every other day) underwent intervention with the palmitoylation inhibitor 2-BP (1 µM). Using the FIJI software and referring to a prior method [25], the transient calcium ion AUC and diffusion distance of neurons were analyzed.

### Statistical analysis

Statistical analyses were performed using IBM SPSS Statistics, version 26 (International Business Machines Corporation, USA). First, we analyzed whether the data were normally distribution. Data that were normally distributed were compared using parametric tests (t tests for two groups and one-way ANOVAs for multi-group comparisons). Data that were not normally distributed were analyzed using a non-parametric test (the Wilcoxon–Mann–Whitney test). *P* < 0.05 was considered statistically significant. Values are presented as the mean ± standard error of the mean (SEM). All measurements and analyses were performed in a blinded manner.

## Results

### Discovery of an FAD pedigree with a novel mutation of the *ZDHHC21* gene

**Fig. 1:**
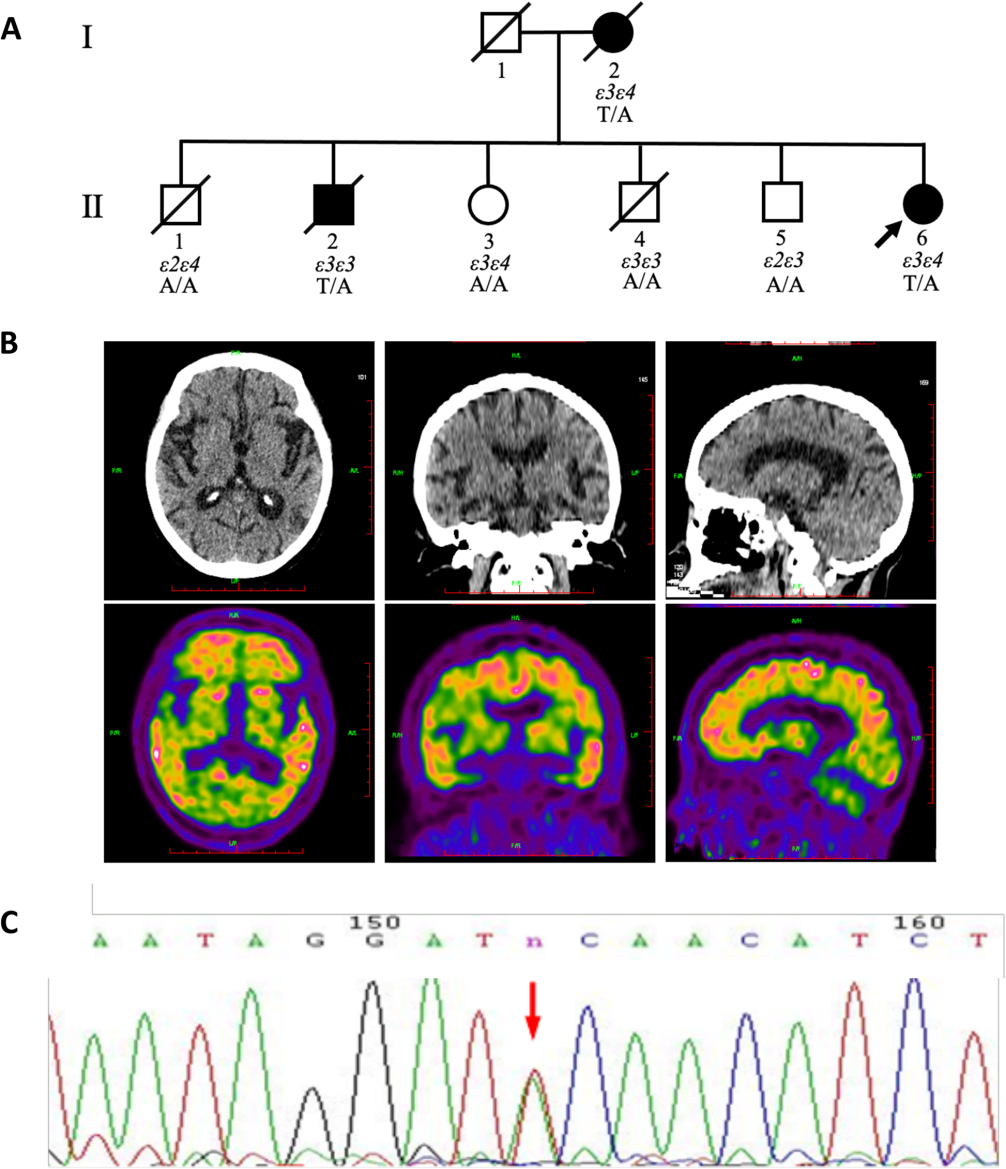
Family information, PET images, and sequencing chromatogram of a *ZDHHC21* mutation in patients with AD. **A** Family pedigree of the patient with the ZDHHC21 p.T209S mutation. The proband is labeled with an arrow, affected family members with black symbols, and unaffected members with white symbols. Men are denoted with squares, women with circles, and deceased members with slashes through the symbols. **B** ^11^C-PIB PET/CT images of the II-6 patient. From left to right: transverse, sagittal, and coronal sections. **C** The sequencing chromatogram of a heterozygous *ZDHHC21* c.999A > T mutation in the patient

The proband (II-6) was a 55-year-old woman (Fig. 1A). The proband presented to our neurology department in 2015 with complaints of gradual and progressive memory decline. Five years prior to that, the patient started experiencing difficulties in remembering what she did in daily life. Two years prior, she was unable to live by herself and needed help from family members. At our memory clinic, she could not answer most of the questions asked by the doctors. Neurological examination revealed cognitive deficits. Her Mini-Mental State Examination (MMSE) score was 5, and her Clinical Dementia Rating (CDR) score was 3. N-methyl-[^11^C]2-(4’-methylaminophenyl)-6-hydroxybenzothiazole (^11^C-PIB) positron emission tomography (PET) revealed considerable Aβ retention in the bilateral frontal, parietal, and lateral temporal cortices. In addition, severe brain atrophy was detected on the corresponding computed tomography (CT) images, especially in the hippocampi. Bilateral MTA score was 3, indicating severe atrophy of the hippocampus (Fig. 1B). In this FAD pedigree, the proband’s mother (I-1) and one brother (II-2) had had AD.

To identify candidate causal gene variants for this FAD pedigree, we performed exome capture followed by deep sequencing. We did not detect any pathogenic mutations known to cause AD, that is, in the *APP*, *PSEN1*, or *PSEN2* genes. The APOE genotypes of all family members were shown in Fig. 1A. The AD patients (II-6, I-2) and two non-AD family members (II-1, II-3) had the APOE ε4 allele, but one AD patient (II-2) was APOE ε4 negative. Further, we identified new candidate causative mutations for this family. Two affected members and two asymptomatic control members were selected for exome sequencing. A high level of coverage (> 70 ×) was achieved, with an average of 7.5 × 10^9^ base pairs sequenced per person. The candidate causative variants were identified and filtered, using the following criteria: the variant present in both affected individuals within the family, not present in the unaffected individual within the family, altered the amino acid sequence, and MAF < 0.5%. Remaining variants were filtered for the next step according to the following rules: CADD > 20; GERP +  +  ≤ 4; SIFT < 0.05; PolyPhen-2 > 0.8; MetaLR > 0.5; and M-CAP > 0.025. Finally, a novel, heterozygous, missense mutation (p.T209S) was identified (Fig. 1C). It was confirmed by Sanger sequencing and tested for Mendelian segregation in all affected family members. The mutation was located in exon 6 of the *ZDHHC21* gene. This mutation had not previously been reported in either the Genome Aggregation Database (gnomAD), DisGeNET, AD and Frontotemporal Dementia (FTD) Mutation Database [26] or The Human Gene Mutation Database (HGMD [27]). Additionally, the pathogenicity of the mutation was predicted using dbSNP [28] and the Exome Variant Server [29]. Evolutionary conservation analysis revealed that the missense mutation p.T209S resulted in a change of a highly conserved amino acid.

### ZDHHC21 mutation induces cognitive decline and aberrant palmitoylation of APP and FYN in mice

**Fig. 2:**
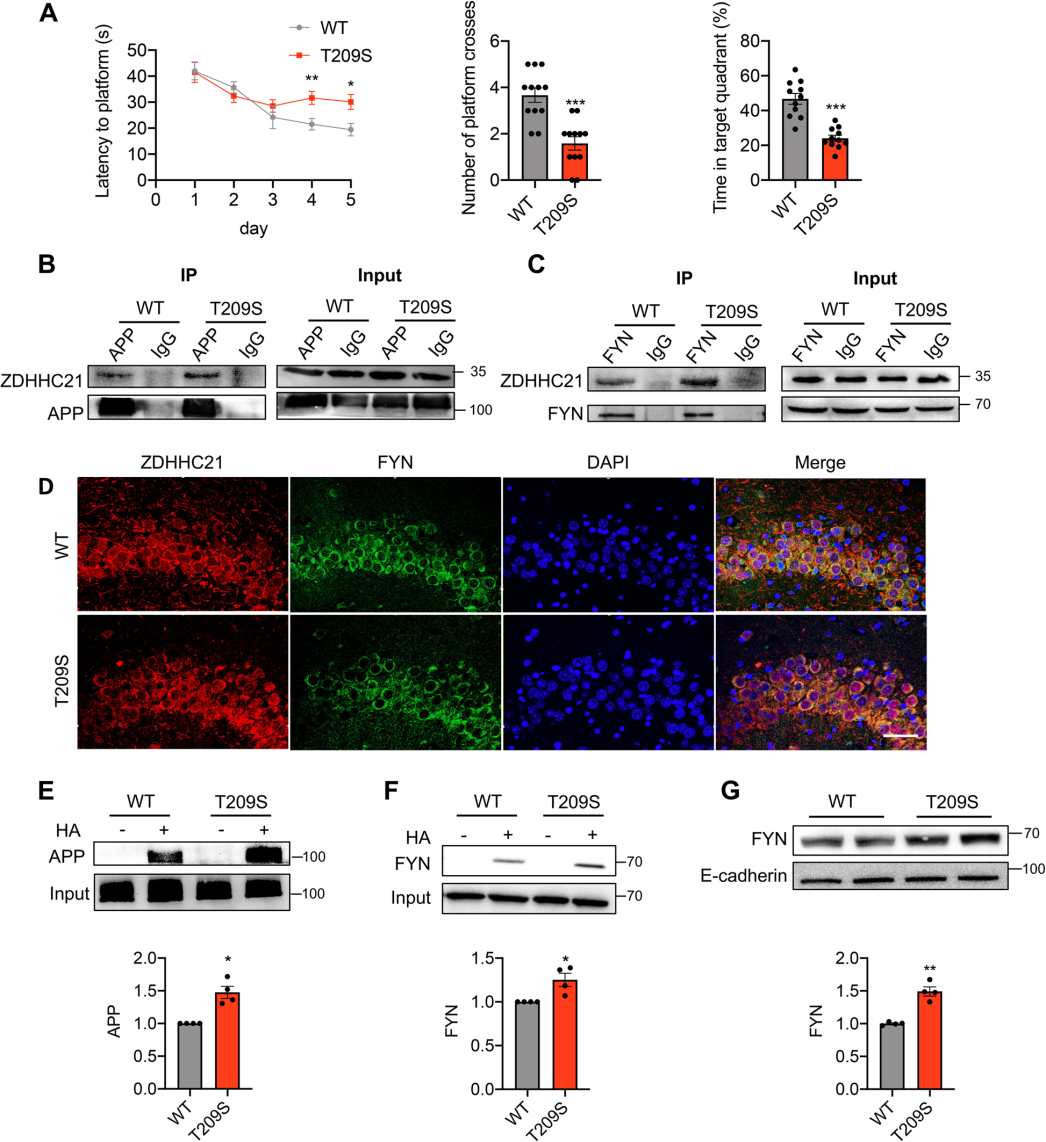
*ZDHHC21* mutation inducing cognitive decline and aberrant palmitoylation of APP and FYN in mice. **A** The Morris water maze test of WT and ZDHHC21^T209S/T209S^ mice (9 months old), including latency to platform (left), number of platform crosses (middle), and time in the target quadrant (right). **B** and **C** Immunoblot showing co-immunoprecipitation of ZDHHC21 with APP (B) or FYN (C) in the WT and p.T209S hippocampi (9 months old). **D** Immunofluorescence of ZDHHC21, FYN, and DAPI in the WT and p.T209S hippocampi (6 months old). **E** and **F** Representative immunoblot (up) and quantification (down) of APP (**E**) or FYN (**F**) palmitoylation levels in WT and ZDHHC21^T209S/T209S^ hippocampi (6 months old). **G** Representative immunoblot (up) and quantification (down) of FYN levels in hippocampal membrane proteins in WT and ZDHHC21.^T209S/T209S^ mice (6 months old). Values are expressed as means ± SEMs of 8 to 11 (A), 3 (B to D), or 4 (E to G) mice per group. Data in B–G were based on three independent experiments. All mouse groups contained mice of both sexes. Data were analyzed via two-way ANOVA with Tukey’s multiple-comparisons test (A: left), unpaired two-tailed Student’s t-tests (A: middle and right, G), and paired two-tailed Student’s t-test (E and F). *P < 0.05, **P < 0.01. Scale bar, 20 μm (D)

We attempted to clarify the pathogenicity biological consequences of the ZDHHC21 p.T209S mutation by generating ZDHHC21^T209S/T209S^ mice with the CRISPR/Cas9 system, substituting adenine with thymine at position 999 in the endogenous *Zdhhc21* gene (Additional file 1: Fig. S1). First, to examine the effects of the p.T209S mutation on spatial learning and memory, we let WT and ZDHHC21^T209S/T209S^ mice perform a Morris water navigation task. The groups had comparable swimming speeds, which indicated that they had no significant neuromotor differences. Although all ZDHHC21^T209S/T209S^ mice and their WT littermates managed the task after a 5-day training period, a significantly impaired learning ability was observed in ZDHHC21^T209S/T209S^ mice compared with their WT littermates (Fig. 2A). A probe trial was performed after the last training session to evaluate memory impairment in terms of spatial reference. Decreased memory retention was observed in ZDHHC21^T209S/T209S^ mice compared with their WT littermates (using the number of platform location crosses and time in the target quadrant as readouts) (Fig. 2A). These results demonstrated that the p.T209S mutation induces cognitive impairment in mice.

We examined whether the mutation affected the expression of ZDHHC21; however, neither the mRNA nor protein levels were altered in the ZDHHC21^T209S/T209S^ mouse brain (Additional file 1: Fig. S2A, B). APP and the tyrosine protein kinase FYN were previously reported as potential substrates for palmitoylation by ZDHHC21 [30, 31]. Thus, we determined whether and how palmitoylation of APP and FYN facilitated the pathological process of AD. We discovered an interaction between ZDHHC21 and APP or FYN in both the WT and ZDHHC21^T209S/T209S^ hippocampi (Fig. 2B, C). Colocalization of ZDHHC21 and FYN was identified in both WT and ZDHHC21^T209S/T209S^ hippocampi via double immunofluorescence (Fig. 2D). We attempted to clarify whether the p.T209S mutation affects the enzymatic activity of ZDHHC21 by comparing the palmitoylation of APP and FYN in the hippocampi of WT and ZDHHC21^T209S/T209S^ mice via the acyl-biotin exchange (ABE) assay. The palmitoylation levels of APP and FYN were 47.6% and 25.2% higher, respectively, in the ZDHHC21^T209S/T209S^ hippocampus than those in the WT hippocampus (Fig. 2E, F), while the expression levels of APP and FYN did not differ between the groups (Additional file 1: Fig. S2C, D), indicating that ZDHHC21 p.T209S is a gain-of-function variant causing an increase in enzymatic activity. It was reported that Fyn palmitoylation is important for its localization to the membrane. We assessed the effect of FYN palmitoylation on protein trafficking by investigating whether FYN is enriched in the membranes of ZDHHC21^T209S/T209S^ mice. We isolated membrane proteins from the brain tissue homogenate extracts of WT and ZDHHC21^T209S/T209S^ mice. As expected, FYN was more abundant in the membrane of ZDHHC21^T209S/T209S^ mice than in those of WT mice. Quantitative analysis revealed that FYN level in membrane was 15.5% higher in ZDHHC21^T209S/T209S^ mice than those in WT mice (Fig. 2G).

### ZDHHC21 mutation induces Aβ accumulation and tau hyperphosphorylation in mice

**Fig. 3:**
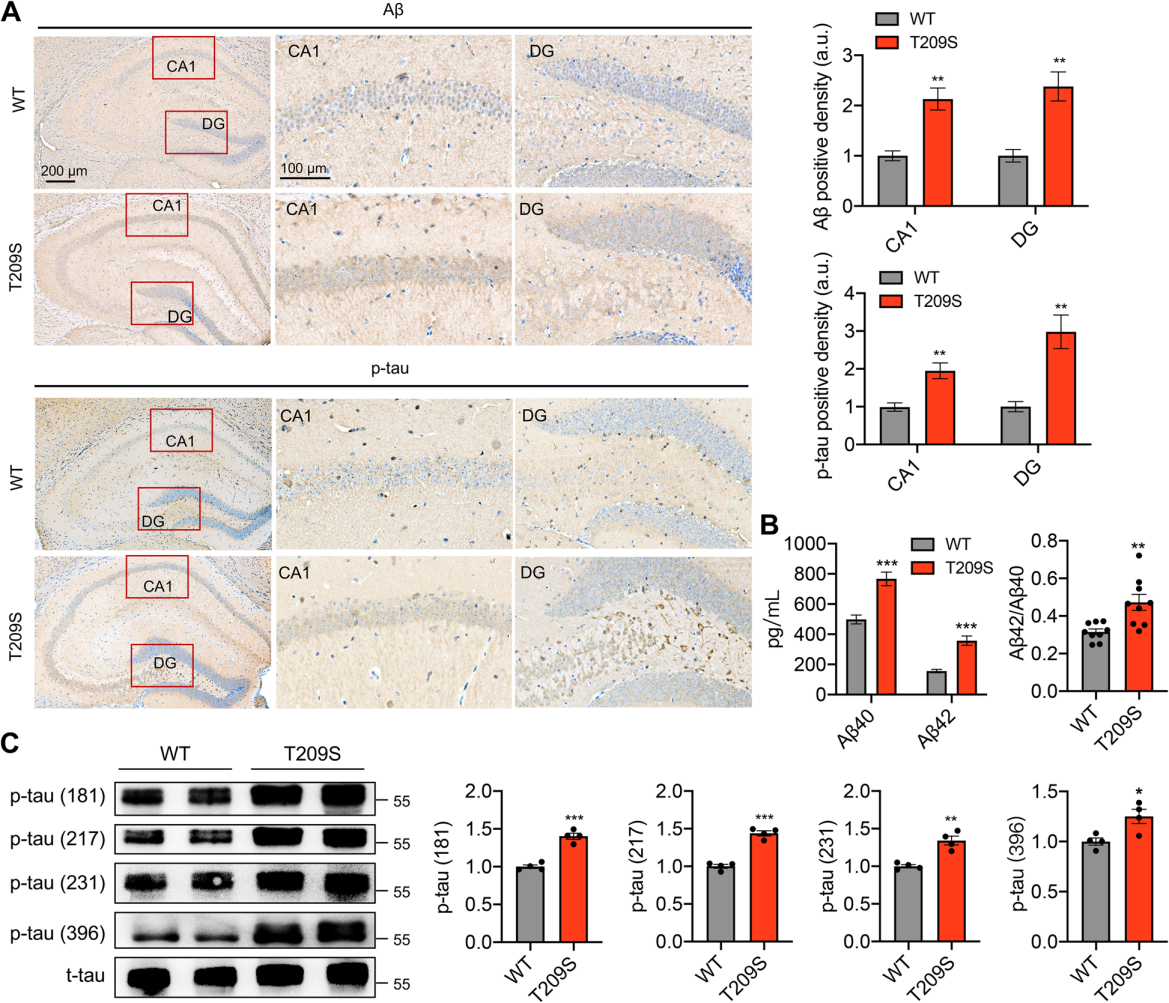
Mutation of ZDHHC21 inducing AD pathologies in mice. **A** Representative immunofluorescence images of Aβ and p-tau in the WT and ZDHHC21^T209S/T209S^ hippocampus (9 months old). **B** Soluble Aβ42 and Aβ40 levels in WT and ZDHHC21^T209S/T209S^ hippocampal tissues (6 months old) were detected using an ELISA. **C** Representative immunoblot (left) and quantification of tau phosphorylation (right) in WT and ZDHHC21.^T209S/T209S^ hippocampi (6 months old). Values are expressed as means ± SEMs of 3 (**A**), 9 (**B**), or 4 (**C**) mice per group. Data are representative of three independent experiments with similar results. All mouse groups consisted of mice of both sexes. Data were analyzed via unpaired two-tailed Student’s t-tests. *P < 0.05, **P < 0.01, ***P < 0.001. Scale bar, 200 μm and 100 μm as indicated, respectively (**A**)

Further, we examined the AD-related pathological changes in the hippocampus of ZDHHC21^T209S/T209S^ mice. Widespread, intraneuronal Aβ and p-tau staining was observed in all regions of the hippocampus in ZDHHC21^T209S/T209S^ mice. Similar patterns have been reported in early-stage APP/PS-1 transgenic mice [32] and an APP Sw-NSE mouse model [33]. Measurement of Aβ levels by using an enzyme-linked immunosorbent assay (ELISA) revealed that Aβ40 and Aβ42 species, which are synaptotoxic in AD, were significantly higher (by 53.0% and 129.7%, respectively) in the hippocampus of ZDHHC21^T209S/T209S^ mice than those in WT mice (Fig. 3B); the ratio of Aβ42 to Aβ40 was higher by 56.7% (Fig. 3B). In addition, the phosphorylation level detected by western blotting was higher in ZDHHC21^T209S/T209^ mice at Thr181, Thr 217, Thr231, and Ser396 in tau (Fig. 3C). Together, these findings demonstrate that the ZDHHC21 p.T209S mutation induces Aβ accumulation and tau hyperphosphorylation in mice.

### ZDHHC21 mutation causes neuronal loss and disturbs synaptic function

**Fig. 4:**
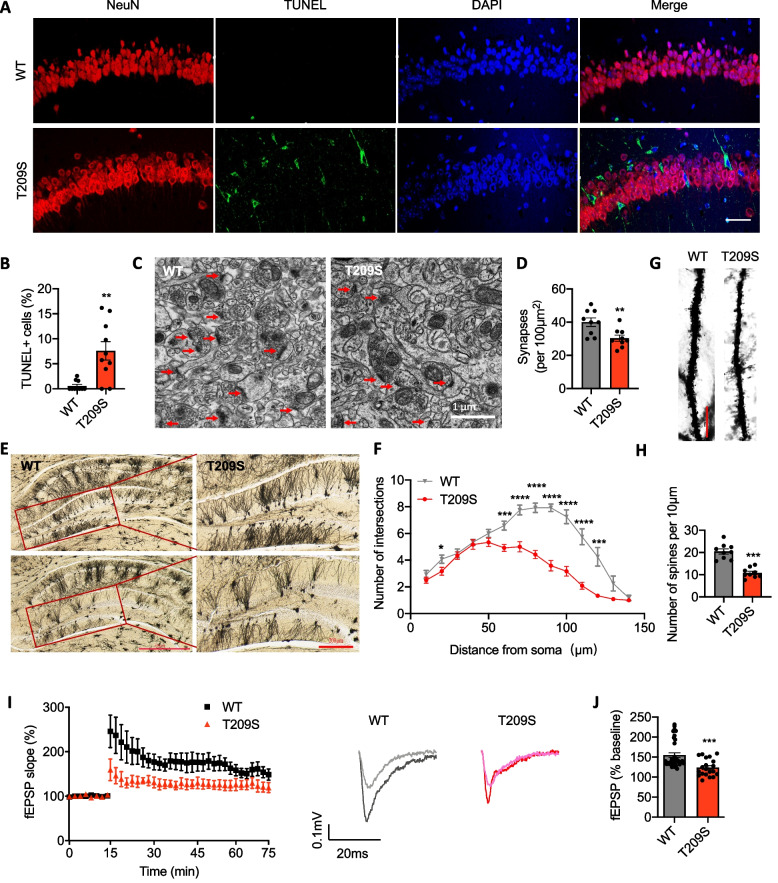
ZDHHC21 mutation leading to neuronal losses and synaptic dysfunction. **A** Immunofluorescence analysis of TUNEL-reactive cells in the CA1 regions of WT and ZDHHC21^T209S/T209S^ hippocampi (9 months old). **B** Quantitative analysis of TUNEL-reactive cells in the CA1 regions of the hippocampus. **C** Representative set of electron microscopy images of WT and ZDHHC21^T209S/T209S^ hippocampi (9 months old). Synapses are indicated with red arrows. **D** Quantification of the number of synapses in (**C**). **E** Representative Golgi stain image of WT and ZDHHC21^T209S/T209S^ hippocampi (9 months old). **F** Quantification of the number of dendritic branches per given distance from the soma. **G** Representative images of Golgi-stained hippocampal pyramidal neurons. **H** Quantification of the number of dendritic spines in (**G**). **I** LTP was induced in acute hippocampal sections from WT and ZDHHC21^T209S/T209S^ mice (3 months old). Exemplary trances in light/dark grey indicate before/after LTP induction for the WT mice, and traces in light/dark red indicate before/after LTP induction for the ZDHHC21^T209S/T209S^ mice. **J** Quantification of fEPSP–50–60 min after induction. Values are expressed as means ± SEMs of 10 sections from 3 mice per group (**B**, **D**), 4 (**F**) and 3 (**H**) neurons from 3 mice per group, or 4 to 7 sections from 3 to 5 mice per group (**J**). Data were analyzed via unpaired two-tailed Student’s t-tests (**B**, **D**, **F**, **H** and **J**). ****P < 0.0001, ***P < 0.001, **P < 0.01, and *P < 0.05. Scale bars, 20 μm (**A**), 1 μm (**C**), 50 μm (**E**: left), 200 μm (**E**: right), and 10 μm (**G**)

We examined hippocampal neuronal apoptosis by using terminal deoxynucleotidyl transferase-mediated dUTP-biotin nick end labeling (TUNEL) staining along with neuronal markers and neuronal nuclei (NeuN). We discovered that the ZDHHC21 p.T209S mutation induced neuronal apoptosis (Fig. 4A, B). Furthermore, compared with WT mice, the density of functional synapses was markedly lower in the hippocampus of ZDHHC21^T209S/T209S^ mice (Fig. 4C, D). We also detected a significantly lower number of dendritic branches between 60 and 120 mm from the soma in mutant than in WT mice, as measured with Sholl analysis (Fig. 4E, F), as well as a smaller number of dendritic spines (Fig. 4G, H). Lastly, to examine the impact of mutations on synaptic plasticity, we performed field recordings of synaptic long-term potentiation (LTP) induced by one train of high-frequency stimuli in the Schaffer collateral pathway. The magnitudes of the field excitatory postsynaptic potentials (fEPSPs) in ZDHHC21^T209S/T209S^ mice were significantly lower (Fig. 4I, J) than those in WT mice, which was consistent with the observed memory impairment. These results indicate that hippocampal neuronal plasticity is impaired in ZDHHC21^T209S/T209S^ mice.

### Synaptic dysfunction of ZDHHC21^T209S/T209^ is mediated via the ZDHHC21-FYN-NMDAR2B pathway

**Fig. 5:**
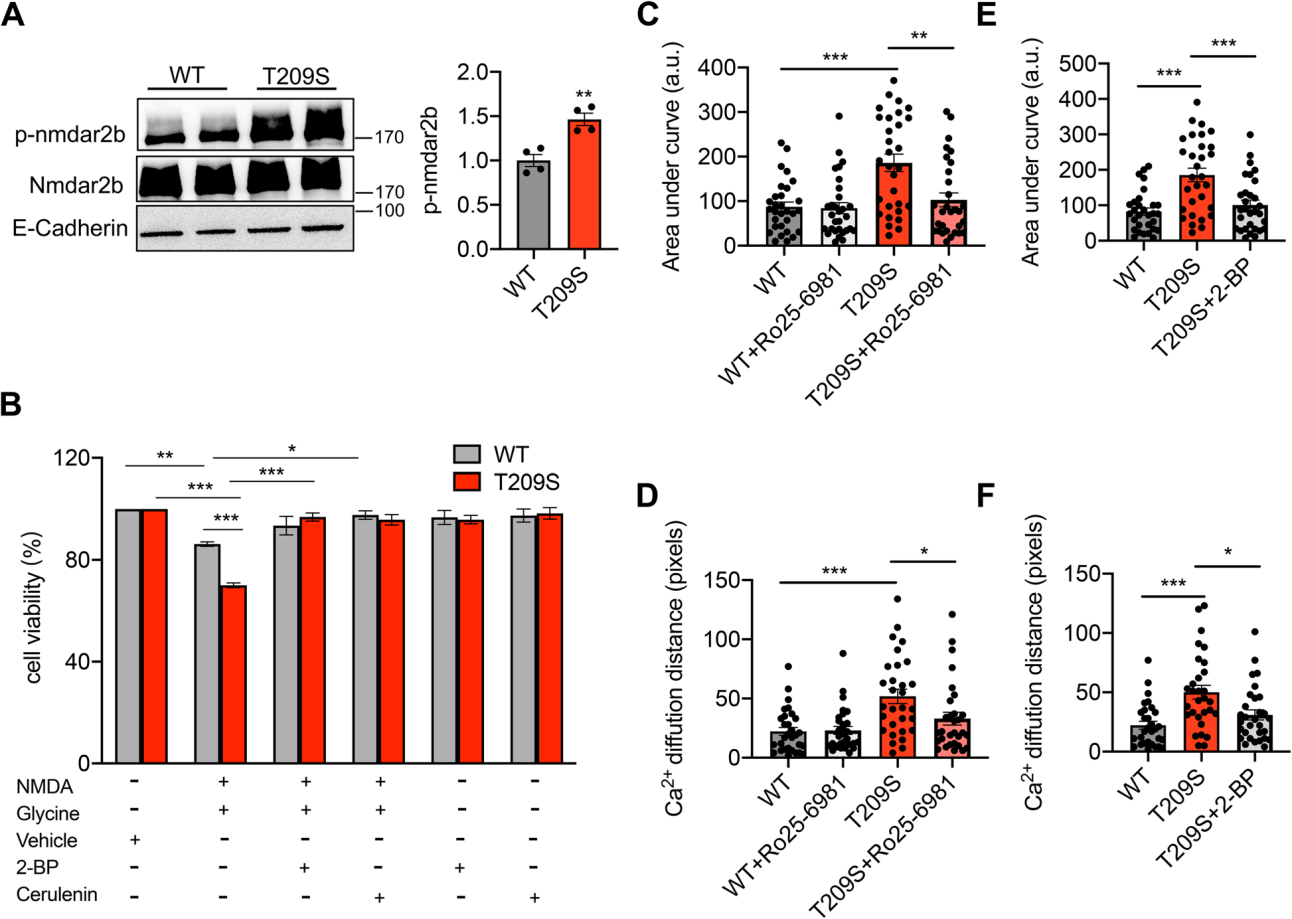
The effect of the ZDHHC21 mutation on synaptic dysfunction via the FYN-NMDAR2B pathway. **A** Representative immunoblot (left) and quantification of the phosphorylation of NMDAR2B (right) in WT and ZDHHC21^T209S/T209S^ hippocampi (mean ± s.e.m.; *n* = 4 mice per group with three independent experiments). **B** Quantification of cellular viability in DIV18-20 WT and p.T209S neurons treated with NMDA and glycine (100/10 μM) with or without pretreatment with vehicle (dimethyl sulfoxide) only, 2-BP (1 μM), or cerulenin (1 μM) (mean ± s.e.m.; *n* = 4 mice per group with three independent experiments). **C** Quantification of the spontaneous calcium transient AUC of WT and p.T209S neurons before and after treatment with Ro25–6981 (mean ± s.e.m.; *n* = 4 mice per group, 30 synaptic sites in total). **D** Quantification of the spontaneous calcium transient diffusion distance of WT and p.T209S neurons before and after treatment with Ro25–6981. **E** Quantification of spontaneous calcium transient AUC in WT, p.T209S, and p.T209S + 2-BP-treated groups. **F** Quantification of spontaneous calcium transient diffusion distance in WT, p.T209S, and p.T209S + 2-BP-treated groups. Values are expressed as means ± SEMs of 4 mice per group with three independent experiments, 30 synaptic sites in total (**C**-**F**). All mouse groups consisted of mice of both sexes. Data were analyzed via one-way ANOVA with Tukey’s post hoc test (**B**), or unpaired two-tailed Student’s t-tests (**A**, **C**, **D**, **E**, and **F**). ***P < 0.001, **P < 0.01, and *P < 0.05

Palmitoylation of proteins affects their subcellular localization and functions [34, 35]. As expected, we observed greater localization of FYN to the cell membrane in the ZDHHC21^T209S/T209S^ hippocampus than in the WT hippocampus (Fig. 2G). FYN was previously reported as a key regulator of N-methyl-d-aspartate receptor (NMDAR) function, which is involved in synaptic plasticity [36]. Subsequently, we assessed the effect of p.T209S on the FYN-NMDAR pathway. The levels of phosphorylated forms of NMDAR subunit epsilon-2 (NMDAR2B) were significantly higher in the ZDHHC21^T209S/T209S^ hippocampus than in the WT hippocampus (Fig. 5A). Excessive NMDAR2B activity can cause excitotoxicity and promote neuronal loss [37]. Upon exposure to 100 μM NMDA, neuronal viability was decreased by 13.8% and 30.0% in WT and p.T209S neurons, respectively (Fig. 5B). These results indicated that p.T209S neurons are more vulnerable to NMDA-mediated excitotoxicity. Thereafter, we investigated whether this increased vulnerability to excitotoxicity resulted from the over-palmitoylation induced by the p.T209S mutation. We treated a subset of neurons with palmitoylation inhibitors (2-bromopalmitate [2-BP] or cerulenin) prior to treatment with NMDA and glycine. Notably, pretreatment with either 2-BP and cerulenin improved the viability of p.T209S neurons similar to that of WT neurons (Fig. 5B). These results indicate that the higher vulnerability of p.T209S neurons to excitotoxicity can be mitigated by correcting palmitoylation.

Furthermore, NMDAR2B over-activated via phosphorylation maintains an open conformation for a prolonged period of time, allowing increased calcium entry per synaptic event [38]. We directly determined the effects of the ZDHHC21 p.T209S mutation on calcium dynamics by treating WT and p.T209S neurons with Ro 25–6981, a potent and selective inhibitor of NMDAR2B, and performing calcium imaging. At baseline, the area under the curve (AUC) of spontaneous calcium signal (Fig. 5C) and the calcium diffusion distance of synapses in p.T209S neurons were higher than those in WT neurons. After Ro 25–6981 treatment, both the AUC (Fig. 5C) and calcium diffusion distance (Fig. 5D) of synapses in p.T209S neurons were rescued to levels comparable to WT levels, while Ro 25–6981 had virtually no effect on the calcium signals recorded in WT cells. These data revealed that p.T209S neurons had stronger synaptic calcium signaling than WT neurons, which was dependent on NMDAR2B overactivity.

We determined whether palmitoylation inhibitors could affect calcium dynamics in p.T209S neurons by pretreating p.T209S neurons with 2-BP before calcium imaging. Notably, treatment with 2-BP significantly decreased the AUCs of the calcium signal in p.T209S neurons to nearly WT levels (Fig. 5E, Additional file 1: Fig. S3). We observed similar changes in the calcium diffusion distance at synaptic sites (Fig. 5F, Additional file 1: Fig. S3). Our data provide support that chronic palmitoylation inhibitor treatment may correct the abnormal palmitoylation level of FYN, repairing synaptic function.

## Discussion

**Fig. 6:**
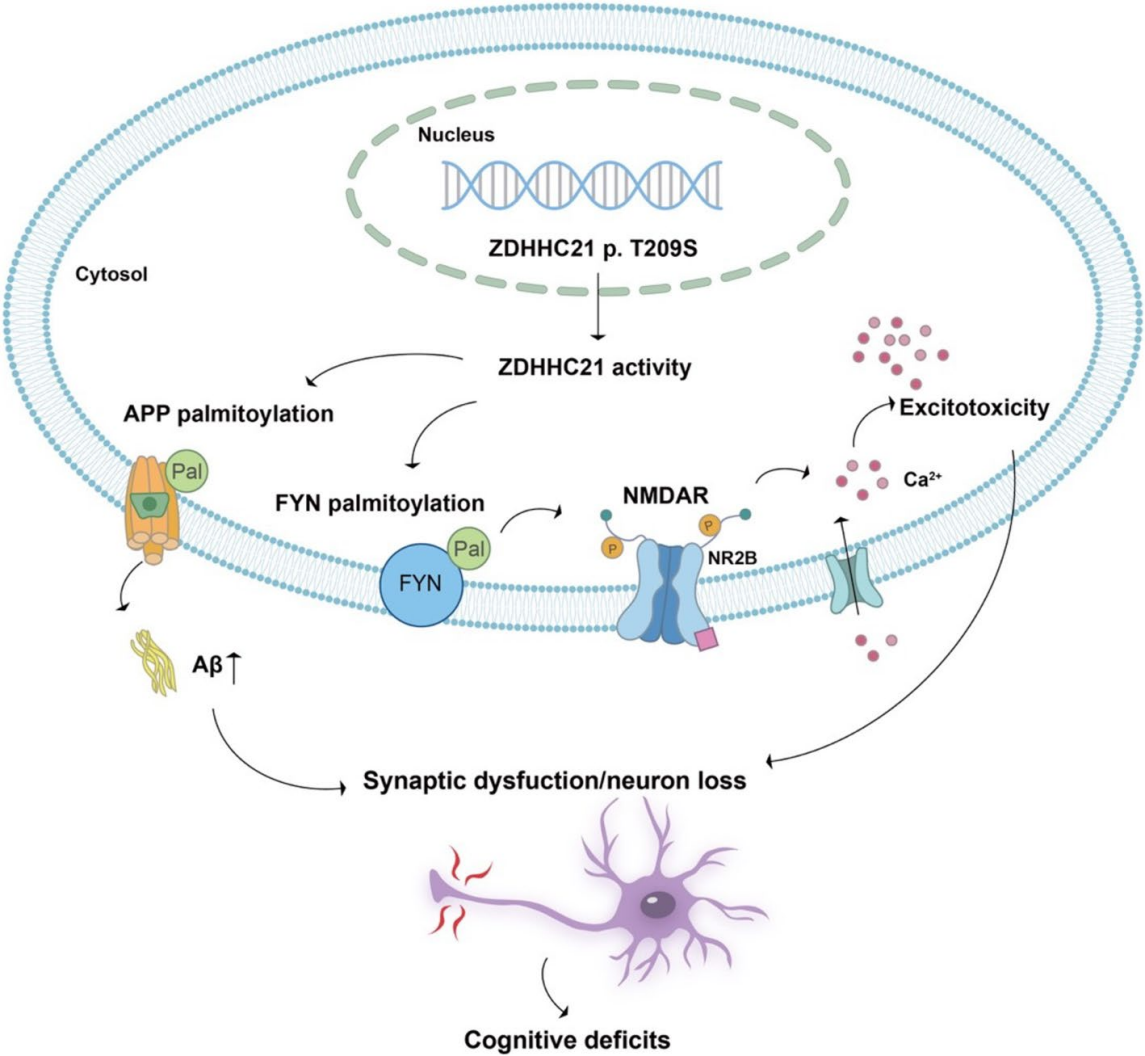
Proposed role of aberrant palmitoylation mediated via ZDHHC21 p.T209S in the development of AD. The ZDHHC21 p.T209S mutation improves enzymatic activity, affects the palmitoylation of APP and FYN, subsequently contributes to the aggravation of the Aβ pathology and synaptic dysfunction, and finally causes cognitive impairment and other AD-related symptoms

In this study, we encountered a family with eight members in two generations, among which the mother and her one son and one daughter were diagnosed with AD, and we confirmed that Aβ exhibited increased retention in the hippocampal, frontal, and parietal areas. Whole exome sequencing revealed a novel and heterozygous missense mutation (p.T209S) in all family members with AD and in none of the unaffected members, indicating cosegregation. All this clinical information supports the family as an AD pedigree. The mutation is located in exon 6 of *ZDHHC21*. This missense mutation had not been previously reported according to the AD and FTD Mutation Database. We aimed to clarify whether it is pathological by generating ZDHHC21^T209S/T209S^ mice with the CRISPR/Cas9 system. In this animal model, we observed aberrant palmitoylation of APP and FYN, increased levels of Aβ and phosphorylated tau (p-tau), disturbed synaptic function, neuronal loss, and memory decline (Fig. 6), suggesting that this mutation may be a candidate causal mutation. In terms of the underlying mechanism, the ZDHHC21-FYN-NMDAR2B pathway played a crucial role in these pathological processes. To the best of our knowledge, this is the first report of a pathological gene mutation other than those in *PSEN1/PSEN2* and *APP* in a Chinese FAD pedigree. Importantly, palmitoylation mediated by the ZDHHC21 p.T209S mutation in the development of AD may provide new insights into the pathogenesis of AD.

In our study, the ZDHHC21 p.T209S mutation enhanced palmitoylation of APP in the hippocampus. Protein palmitoylation plays vital roles in regulating protein stability, subcellular localization, membrane trafficking, and various other cellular processes [30, 39, 40]. Overexpression of ZDHHC21 reportedly increases palmitoylation of APP in PC-12 cells [41]. APP can be processed by BACE1 and γ-secretase, which are both found in membrane microdomains known as detergent-resistant lipid rafts [42, 43]. Palmitoylated APP (palAPP) is preferentially targeted to lipid rafts, where palAPP serves as a better substrate for BACE1 cleavage for Aβ generation than does unmodified APP in vivo [31]. Usually, 10% of APP proteins undergoes post-translational palmitoylation. In the current study, we discovered that the p.T209S mutation enhanced APP palmitoylation, followed by an increase in Aβ40 and Aβ42 levels in the hippocampus of ZDHHC21^T209S/T209S^ mice. Aβ generation has traditionally been thought to involve continuous cleavage of β- and γ-secretase [44, 45]. Accordingly, mutations in *PSEN1*, *PSEN2*, and *APP*, identified in FAD, were reported to increase Aβ production by regulating the catalytic activity and/or aberrant expression of proteins [46]. The proteolytic events resulting in Aβ production are well characterized [47], but do not explain the mechanism in pedigrees without *PSEN1/PSEN2* or *APP* mutations. In our study, ZDHHC21 p.T209S, the first probable pathological mutation identified in a Chinese FAD pedigree (apart from those in *PSEN1/PSEN 2* and *APP*), caused an increase in Aβ accumulation in the mouse brain by enhancing the palmitoylation of APP, which occurred before β- and γ-secretase cleavage of APP. This indicates that the modification of APP, which led to an increase in Aβ production in the present study, was different from the mechanism previously reported in FAD.

In this study, FYN hyperpalmitoylation was identified in ZDHHC21^T209S/T209S^ mice, implying that this mutation disturbed the palmitoylation activity of ZDHHC21. FYN is a direct palmitoylation target of ZDHHC21, and its palmitoylation determines its localization and function in multiple signaling pathways [48]. A previous study demonstrated that mutation of the depalmitoylating enzyme palmitoyl-protein thioesterase 1 (PPT1) causes FYN hyperpalmitoylation, which impairs synaptic transmission and plasticity [25, 49]. Our results also revealed that FYN hyperpalmitoylation induced by the p.T209S mutation reduced the number of synapses and impaired synaptic plasticity. NMDAR2B was previously reported as a major target of FYN [50, 51]. FYN phosphorylates NMDAR2B, increasing NMDAR2B trafficking and membrane stabilization, which results in abnormal synaptic transmission and plasticity [52–54]. Overactivation of NMDARs causes excitotoxicity and neuronal cell damage, and chronic NMDAR hyperactivity contributes to neuronal loss in the development of AD [37, 55] We demonstrated that hyperpalmitoylated FYN increases NMDAR phosphorylation, resulting in ZDHHC21^T209S/T209S^ neurons being more susceptible to NMDA-induced excitotoxicity of postsynaptic receptors, similar to that reported in *Ppt1*^*−/−*^ neurons [25]. In addition, an age-related increase in palmitoylated FYN in the frontal cortex is reportedly associated with poorer reference memory and/or executive functions, suggesting that a perturbation in palmitoylation may contribute to age-related cognitive decline [56]. We also observed that learning and memory were seriously impaired in ZDHHC21^T209S/T209S^ mice, suggesting that activation of FYN signaling induced by the ZDHHC21 mutation was closely associated with cognitive decline.

It would be interesting to determine whether ZDHHC21 is a useful target in AD therapeutics, as elevated levels of palmitoylation seem to have a detrimental role in synaptic integrity and function in AD. Notably, in this study, the chronic palmitoylation inhibitors 2-BP and cerulenin mitigated the increased sensitivity and the impaired synaptic function of ZDHHC21^T209S/T209S^ neurons, which suggested that maintenance of a palmitoylation balance may be used as therapeutic strategy for AD. In the future, animal experiments should be conducted to explore the therapeutic effects of palmitoylation inhibitors on AD mice.

In the present study, a novel, heterozygous missense mutation (ZDHHC21 p.T209S) was detected by whole exome sequencing in an FAD pedigree. This mutation was detected in all family members with AD, while it was not present in unaffected members, indicating cosegregation and a possible pathogenicity. To clarify the pathogenicity of ZDHHC21 T209S mutation in AD, ZDHHC21^T209S/T209S^ mice were generated. This model showed reduced spatial learning function, impaired synaptic function, and increased Aβ production and tau phosphorylation. This mouse model may not completely replicate the pathology of AD in humans because of the absence of Aβ plaques. In existing AD mouse models, mice with only PSEN or MAPT gene mutations did not produce Aβ plaques; the same was true for mice with single APP mutations, such as APP E693Δ-Tg, APP Dutch, and APP Sw-NSE [33, 57, 58]. Based on the analysis of AD mouse models and the findings of this study, we suspect that human APP and multiple mutations may facilitate the development of Aβ plaques; however, this requires more exploration. Although the ZDHHC21 T209S mutation was identified in a Chinese FAD pedigree, its relevance to AD morbidity in the population should be further studied in the future.

## Conclusions

In conclusion, we have provided multiple lines of supporting evidence that the ZDHHC21 p.T209S mutation contributes to AD by enhancing the palmitoylation of APP and FYN, impairing the synapses, and aggravating Aβ pathology, tau hyperphosphorylation, and neuronal loss, ultimately causing cognitive impairment in mice. Our results indicate that modified palmitoylation of ZDHHC21-specific substrates is an alternative mechanism driving AD and a potential target for drug therapy.

## Supplementary Information

Additional file 1: **Fig S1.**Construction of ZDHHC21^T209S/T209S^ mice. **Fig S2.** The expression of ZDHHC21, APPand FYNin WT and ZDHHC21^T209S/T209S^ mice. **Fig S3.** Representative tracesat one synaptic site fromWT, T209Sand T209S+2-BPneurons.

Additional file 2: **Figure S1.**The original blot images of ZDHHC21 and APP in Figure 2B. **Figure S2.** The original blot images of ZDHHC21 and FYN in Figure2C. **Figure S3.** The original blotimages of APP in Figure 2E. **Figure S4.**The original blot images of FYN in Figure 2F. **Figure S5.** The original blot images of FYN and E-cadherin in Figure2G. **Figure S6.** The original blotimages of P-tau, P-tau, P-tau, T-tau and Actin in Figure 3C. **Figure S7.** The original blot images ofP-NMDAR2B, NMDAR2B and E-cadherin in Figure 5A.

## Data Availability

The datasets supporting the conclusions of this article are included within the article and its additional files.

## Abbreviations

AD: Alzheimer’s disease
ABE: Acyl-biotin exchange
Aβ: Amyloid beta
APP: Aβ precursor protein
AUC: Area under the curve
2-BP: 2-Bromopalmitate
CFAN: Chinese Familial Alzheimer’s Disease Network
CDR: Clinical Dementia Rating
^11^C-PIB: N-methyl-[^11^C]2-(4’-methylaminophenyl)-6-hydroxybenzothiazole
CT: Computed tomography
ELISA: Enzyme-linked immunosorbent assay
FAD: Familial Alzheimer’s disease
fEPSPs: Field excitatory postsynaptic potentials
FTD: Frontotemporal Dementia
LTP: Long-term potentiation
MMSE: Mini-Mental State Examination
NeuN: Neuronal nuclei (NeuN)
NMDAR: N-methyl-d-aspartate receptor
NMDAR2B: NMDAR subunit epsilon-2
PET: Positron emission tomography
PPT1: Palmitoyl-protein thioesterase 1
PSEN1: Presenilin-1
PSEN: Presenilin-2
p-tau: Phosphorylated tau
TUNEL: Terminal deoxynucleotidyl transferase-mediated dUTP-biotin nick end labeling
ZDHHC21: Zinc finger DHHC-type palmitoyltransferase 21
