# Supplementary material for: Aberrant palmitoylation caused by a ZDHHC21 mutation contributes to pathophysiology of Alzheimer’s disease
Source: BMC Med. 2023 Jun 26;21:223. doi: 10.1186/s12916-023-02930-7 (PMC10294511; doi:10.1186/s12916-023-02930-7)
Supplement: Supplementary file 1 — Additional file 1: Fig S1.Construction of ZDHHC21T209S/T209S mice. Fig S2. The expression of ZDHHC21, APPand FYNin WT and ZDHHC21T209S/T209S mice. Fig S3. Representative tracesat one synaptic site fromWT, T209Sand T209S+2-BPneurons. [file 12916_2023_2930_MOESM1_ESM.pdf]

Supplementary Figure 1. Construction of ZDHHC21$^{T209S/T209S}$ mice.

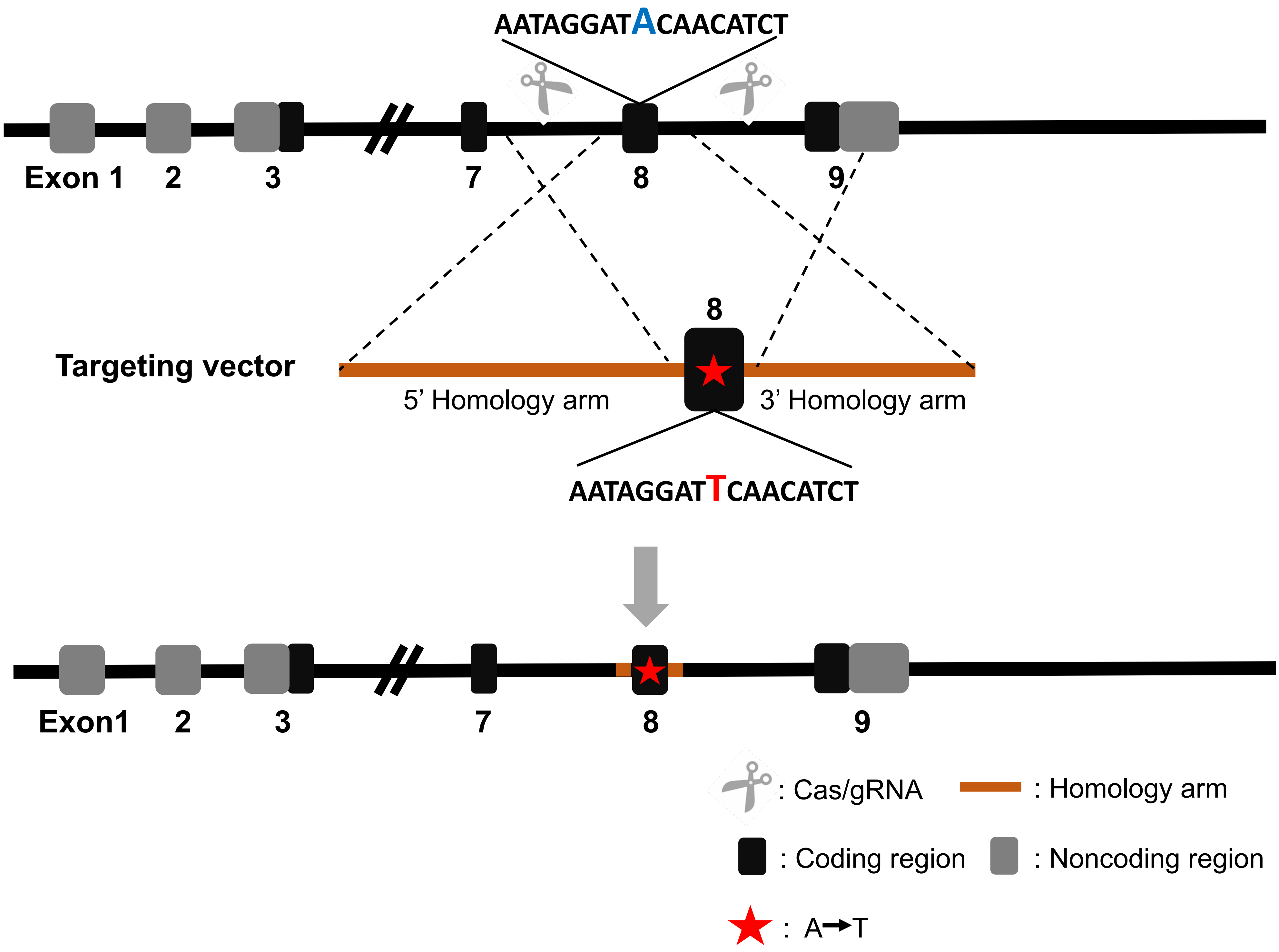

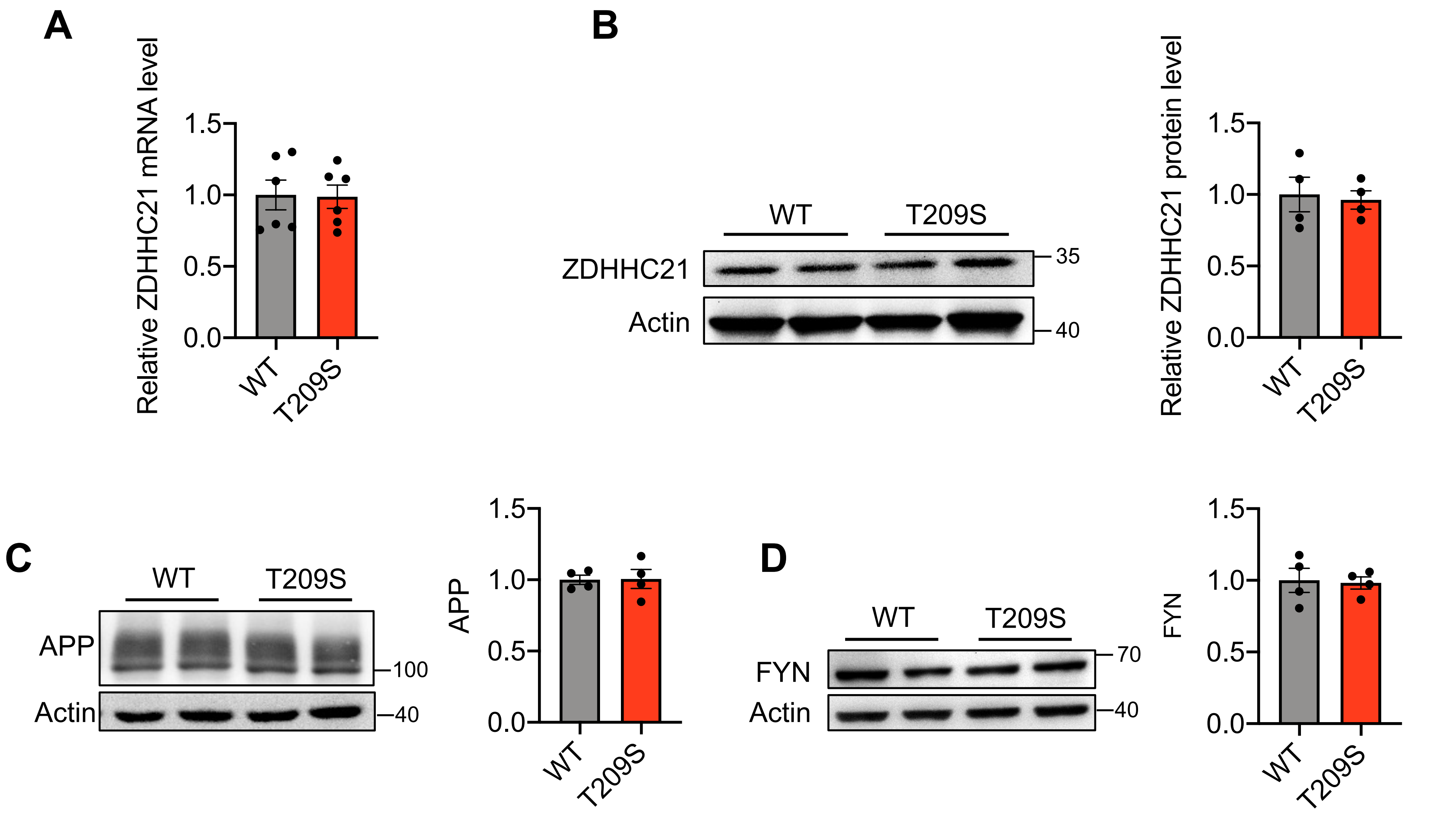

Supplementary Figure 2. The expression of ZDHHC21 (A and B), APP (C) and FYN (D) in WT and ZDHHC21$^{T209S/T209S}$ mice. n = 6 mice per group (a), 4 mice per group (b-d); unpaired two-tailed Student's t-tests. Bars represent the mean and error bars represent the SEM.

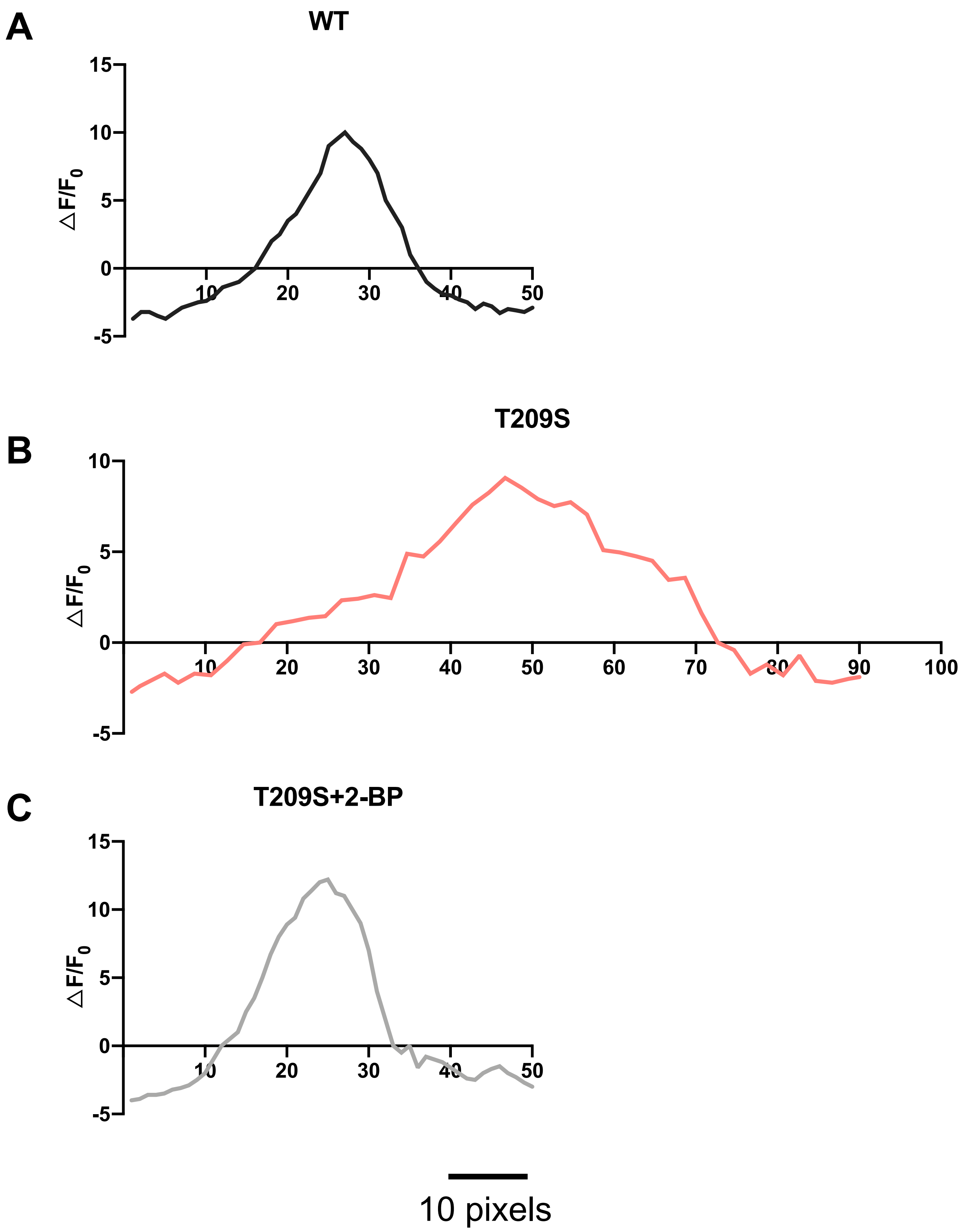

Supplementary Figure 3. Representative traces (averaged $\triangle F/F_0$ responses) at one synaptic site from WT (A) , T209S (B) and T209S+2-BP (C) neurons.
