# Supplementary material for: Aberrant palmitoylation caused by a ZDHHC21 mutation contributes to pathophysiology of Alzheimer’s disease
Source: BMC Med. 2023 Jun 26;21:223. doi: 10.1186/s12916-023-02930-7 (PMC10294511; doi:10.1186/s12916-023-02930-7)

Figure S1. The original blot images of ZDHHC21 and APP in Figure 2B

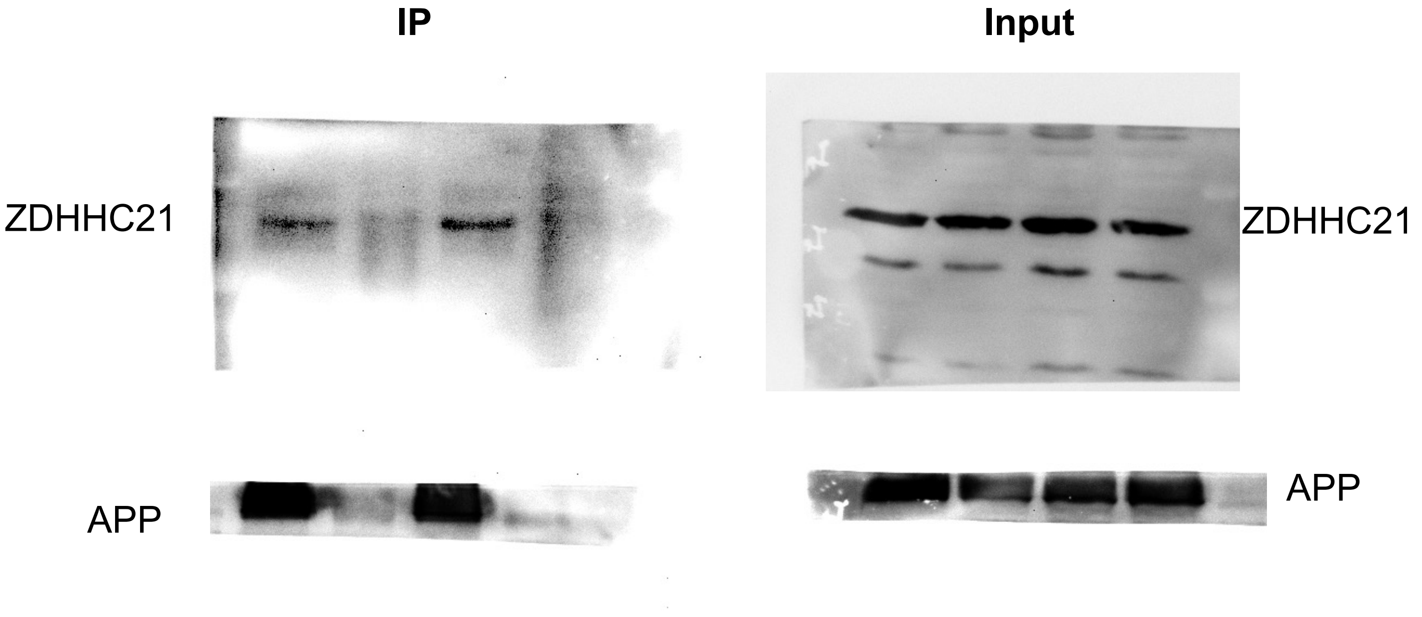

Figure S2. The original blot images of ZDHHC21 and FYN in Figure 2C

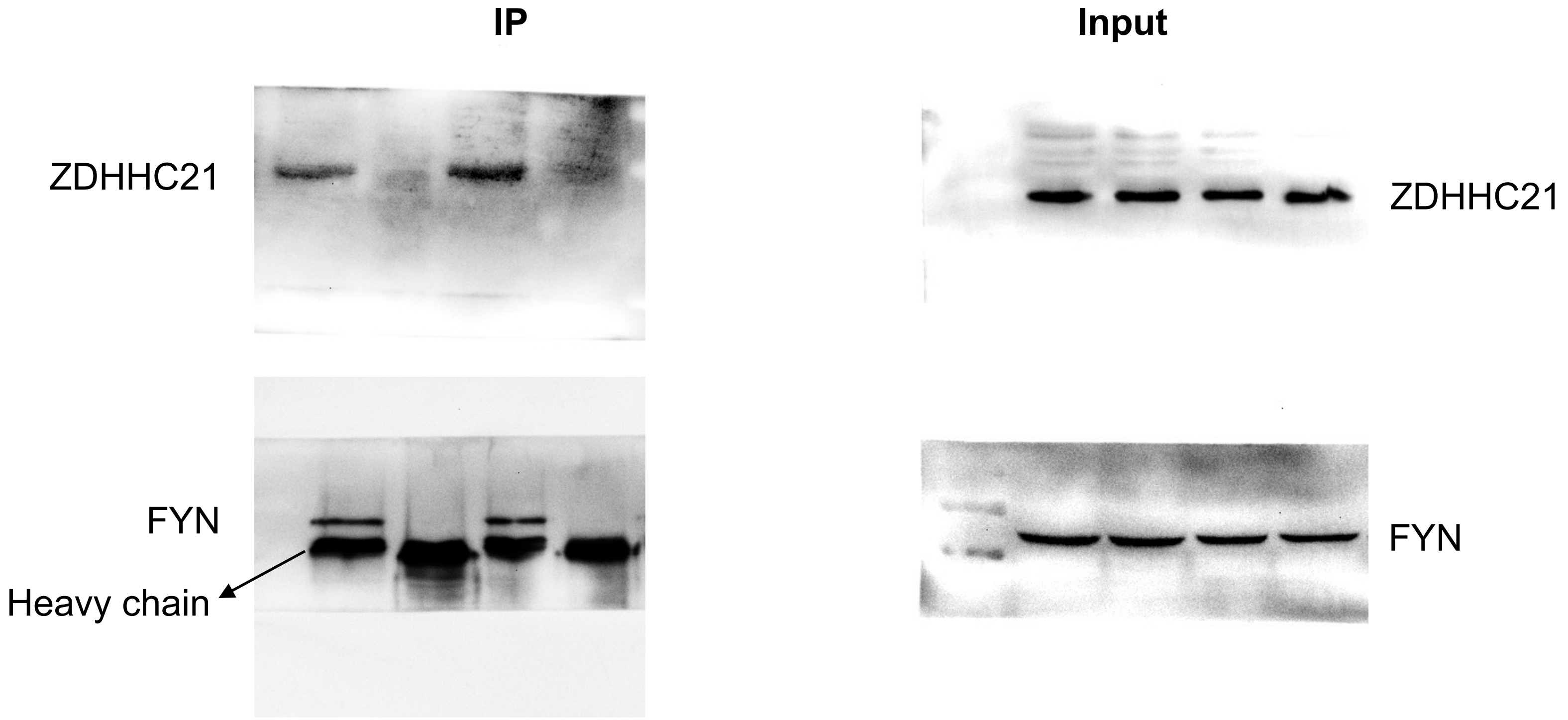

Figure S3. The original blot images of APP in Figure 2E

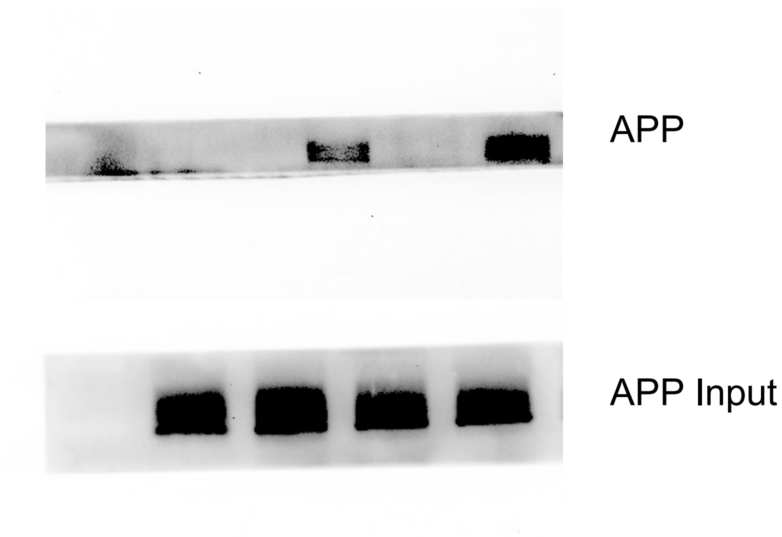

Figure S4. The original blot images of FYN in Figure 2F

FYN

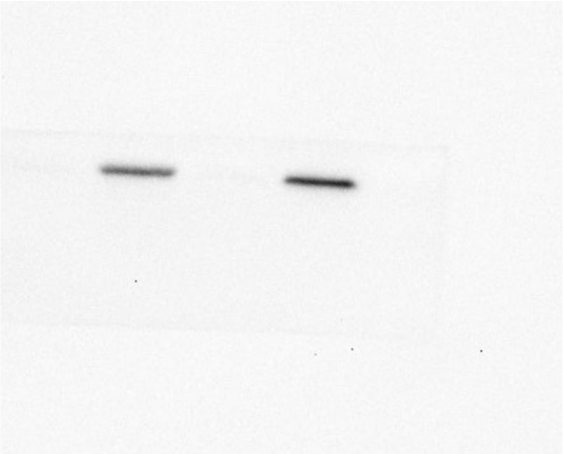

FYN Input

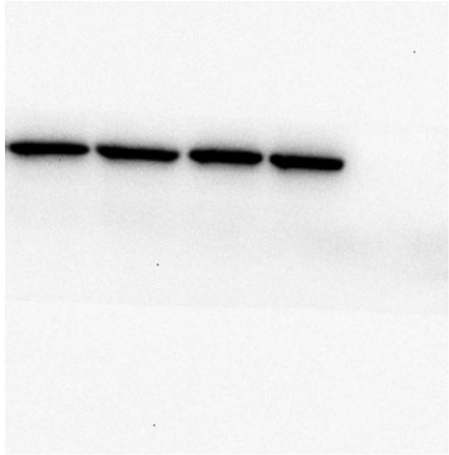

Figure S5. The original blot images of FYN and E-cadherin in Figure 2G

FYN

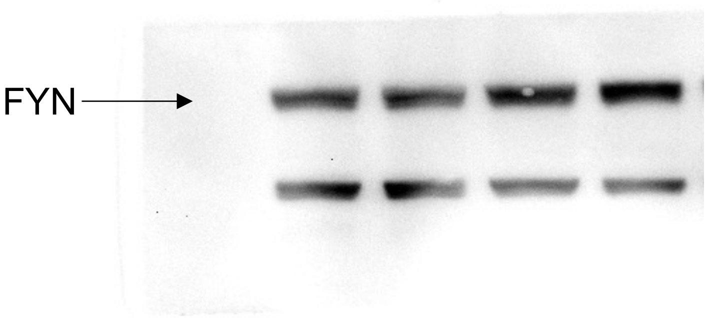

E-cadherin

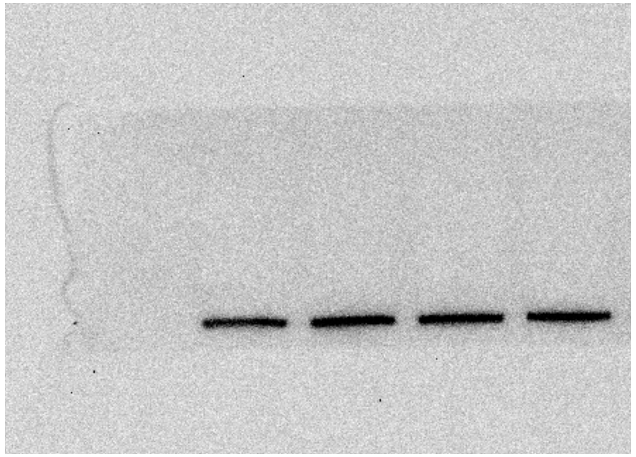

Figure S6. The original blot images of P-tau(396), P-tau(181) , P-tau(231), T-tau and Actin in Figure 3C

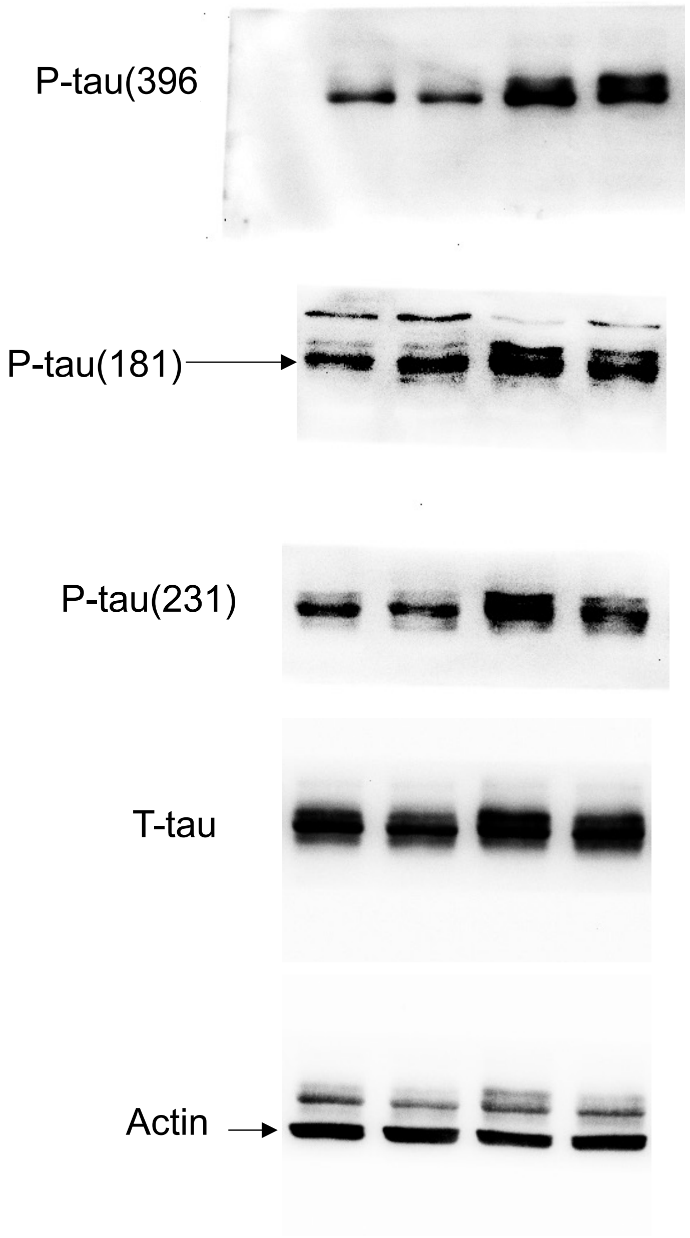

Figure S7. The original blot images of P-NMDAR2B, NMDAR2B and E-cadherin in Figure 5A

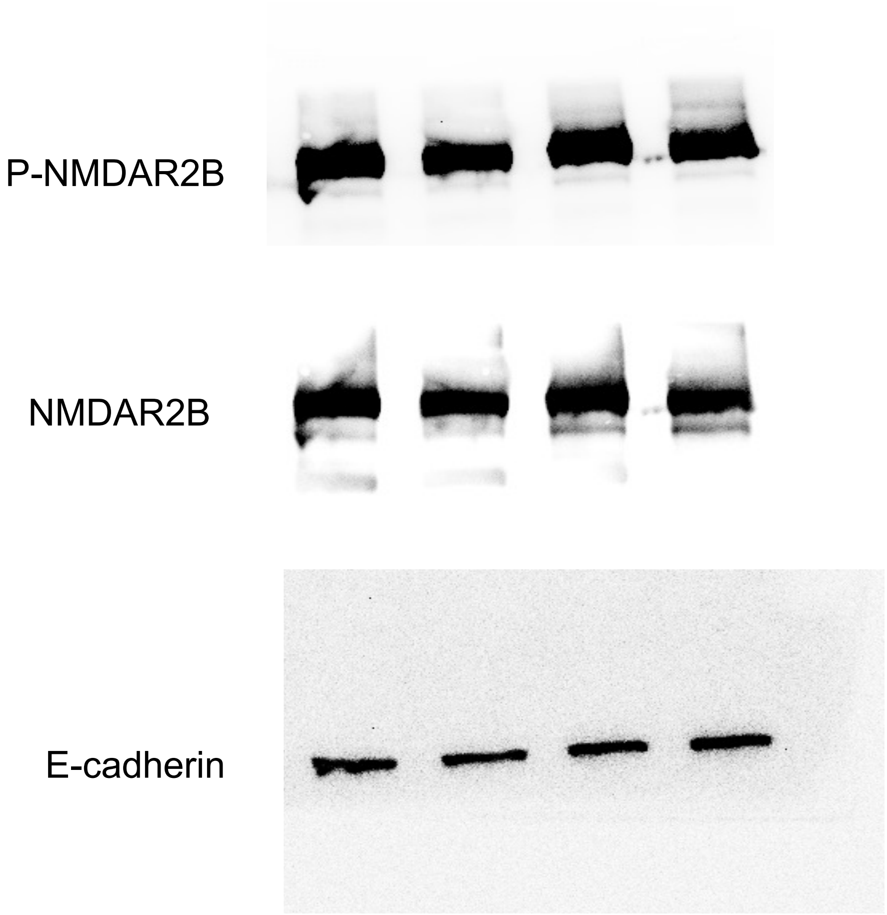

Supplement: Supplementary file 2 — Additional file 2: Figure S1.The original blot images of ZDHHC21 and APP in Figure 2B. Figure S2. The original blot images of ZDHHC21 and FYN in Figure2C. Figure S3. The original blotimages of APP in Figure 2E. Figure S4.The original blot images of FYN in Figure 2F. Figure S5. The original blot images of FYN and E-cadherin in Figure2G. Figure S6. The original blotimages of P-tau, P-tau, P-tau, T-tau and Actin in Figure 3C. Figure S7. The original blot images ofP-NMDAR2B, NMDAR2B and E-cadherin in Figure 5A. [file 12916_2023_2930_MOESM2_ESM.pdf]
